# Supplementary figures and images for: Crystal structure of human Acinus RNA recognition motif domain
Source: PeerJ. 2018 Jul 4;6:e5163. doi: 10.7717/peerj.5163 (PMC6057467; doi:10.7717/peerj.5163)

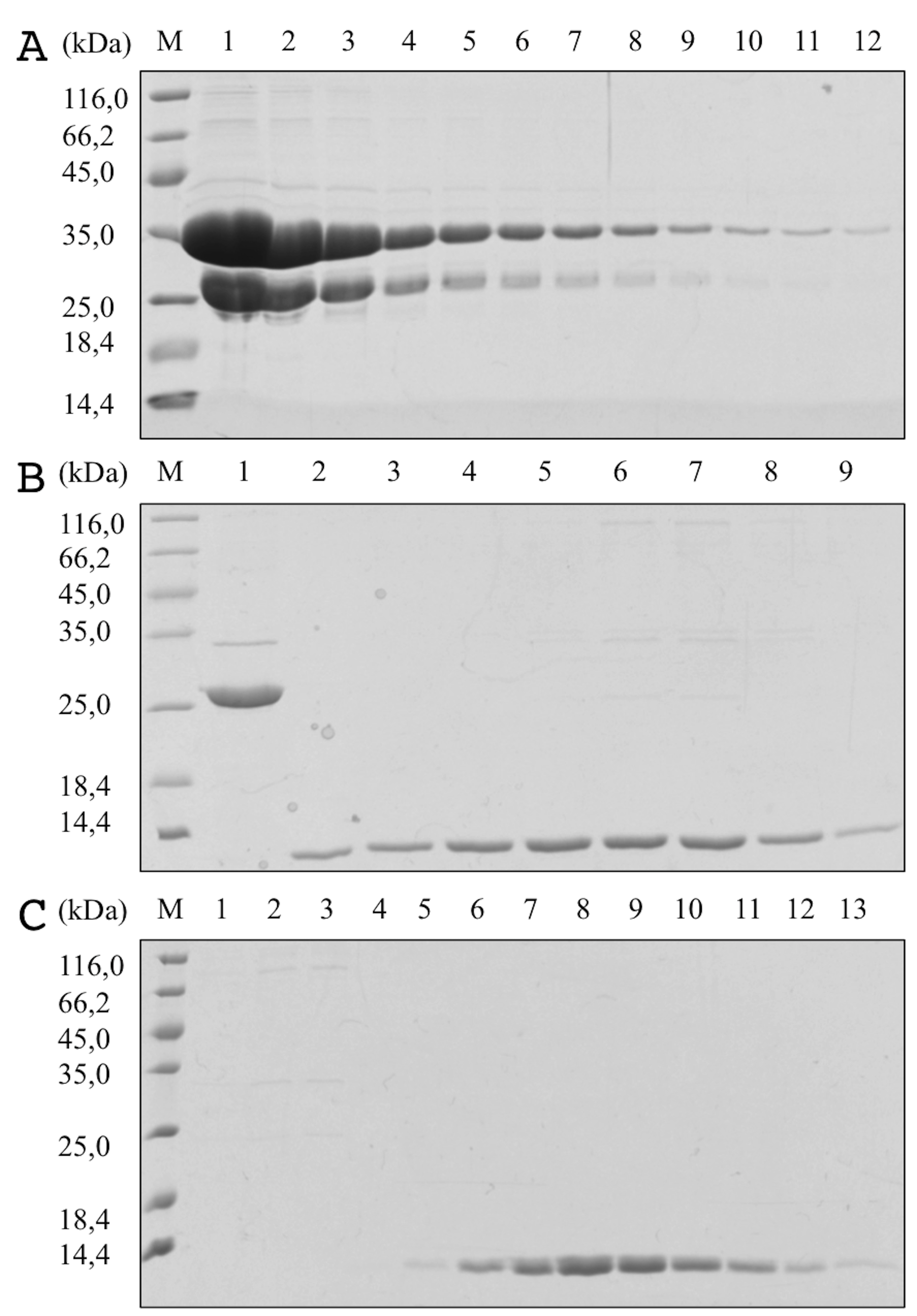

Supplement: Supplemental Information 1 — (A) 1-12–fractions of GST-AcRRM after purification by affinity chromatography using glutathione-agarose; (B) fractions after protease cleavage and purification by heparin chromatography, 1–flow through, 2-9–fractions of AcRRM; (c) 1-13–fractions after size-exclusion chromatography. Fractions 6 to 12 were joined, concentrated and used for crystallization. Lane M contains molecular-mass marker (labelled in kDa). [file peerj-06-5163-s001.png]

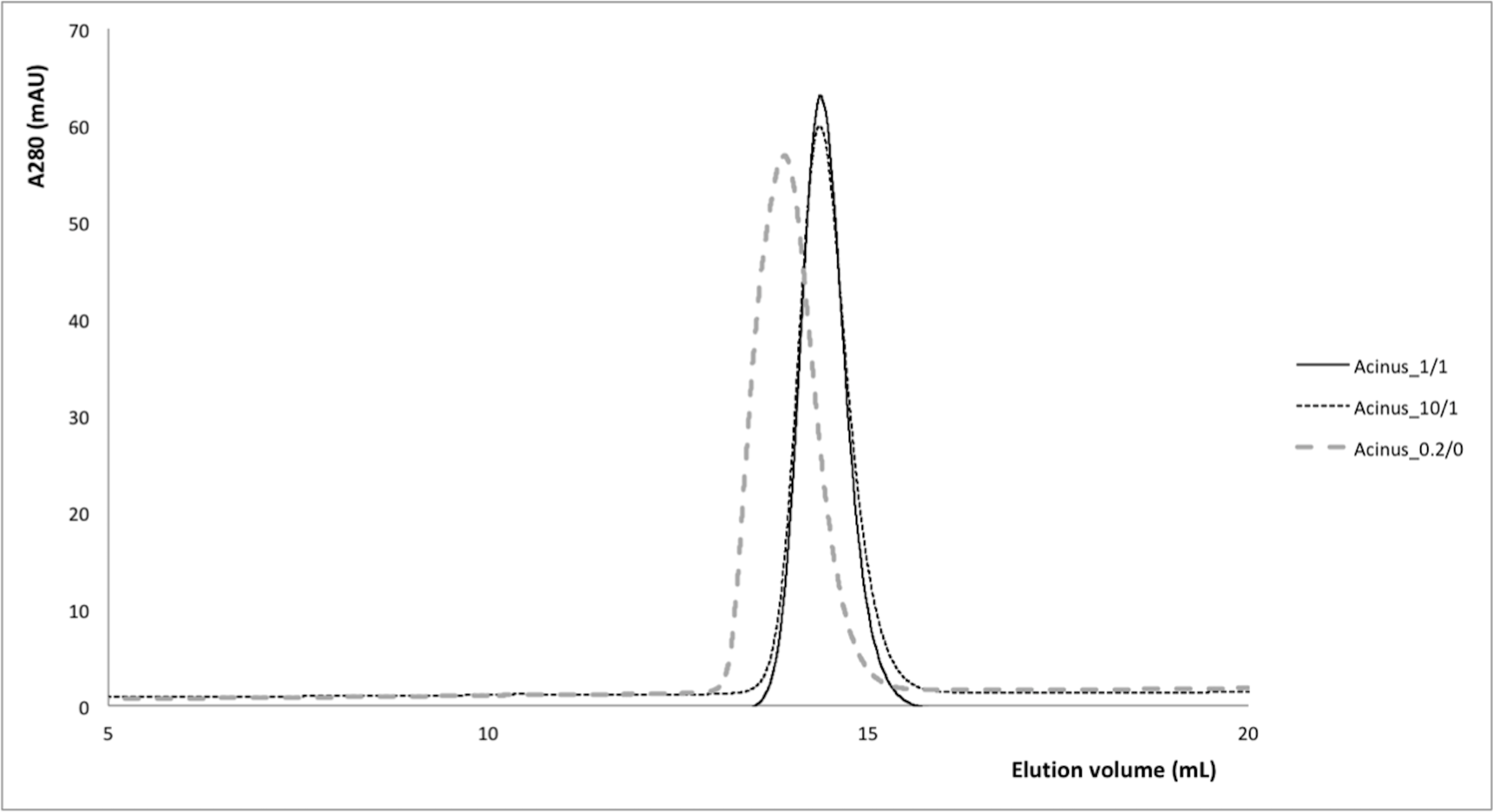

Supplement: Supplemental Information 2 — For all experiments a Superdex 75 10/300 GL column was pre-equilibrated with 50 mM Hepes-NaOH pH 8, 100 mM NaCl buffer and DTT as detail for each case. Black curve is for AcRRM in 1 mM DTT separated on a column pre-equilibrated with buffer supplemented with 1 mM DTT. Doted black cure is for AcRRM in 10 mM DTT separated on a column pre-equilibrated with buffer supplemented with 1 mM DTT. Dashed grey curve is for AcRRM in 0.2 mM DTT separated on a column pre-equilibrated with buffer without DTT. [file peerj-06-5163-s002.png]

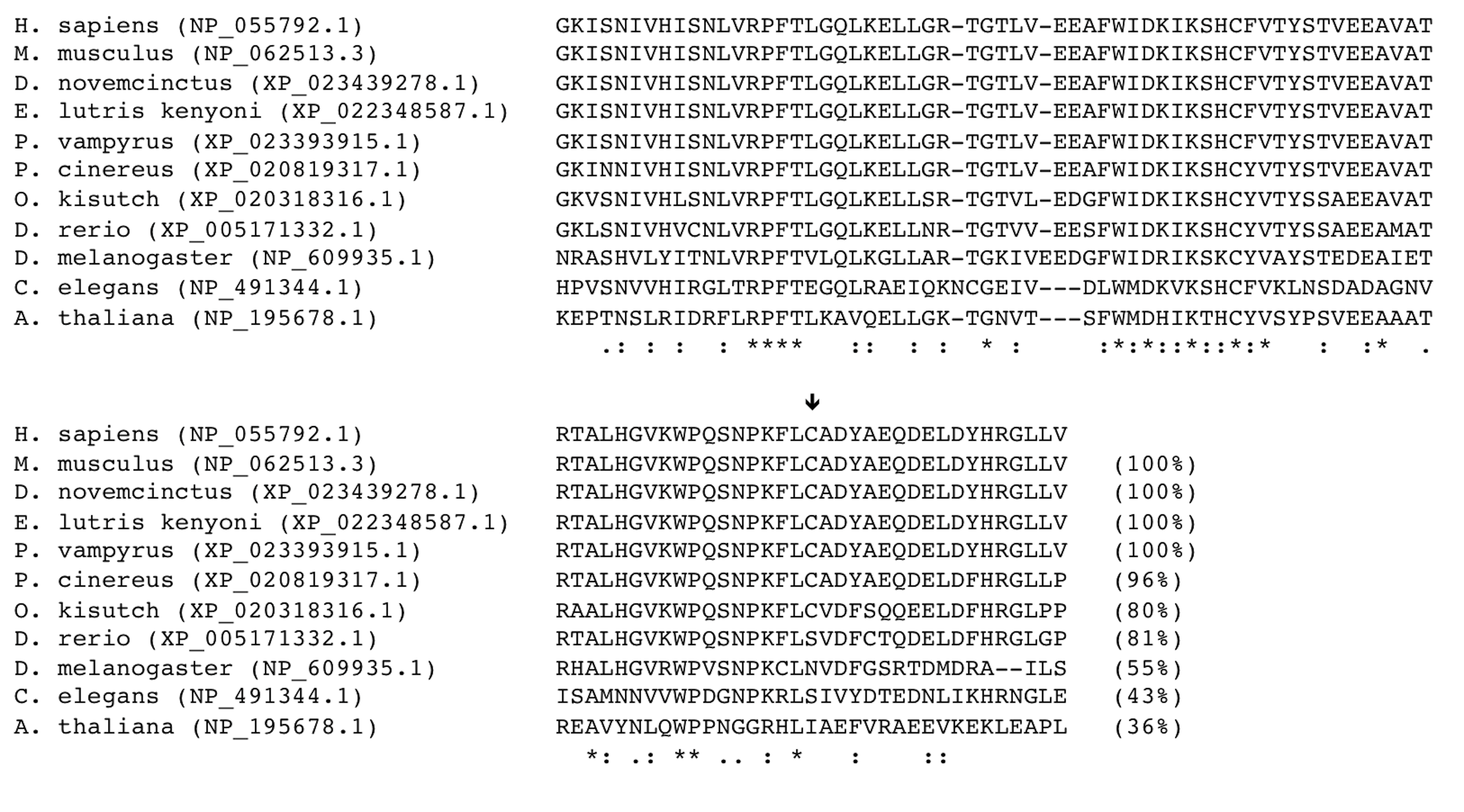

Supplement: Supplemental Information 3 — Sequence alignment of the RRM domain of Acinus homologues from the following species: Homo sapiens, Mus musculus, Dasypus novemcinctus, Enhydra lutris kenyoni, Pteropus vampyrus, Phascolarctos cinereus, Oncorhynchus kisutch, Danio rerio, Drosophila melanogaster, Caenorhabditis elegans, and Arabidopsis thaliana. Sequence IDs are given in brackets, and sequence identity to the human homologue are shown after each sequence. An arrow indicated the position of C1083 (of the human homologue). Alignment analysis was performed using Clustal Omega (Sievers & Higgins, 2017). Fifteen residues identical in all proteins are highlighted with asterisks (*). The conserved substitutions with similar characteristics and semi-conserved substitutions are labelled with (:) and (.), respectively. [file peerj-06-5163-s003.png]

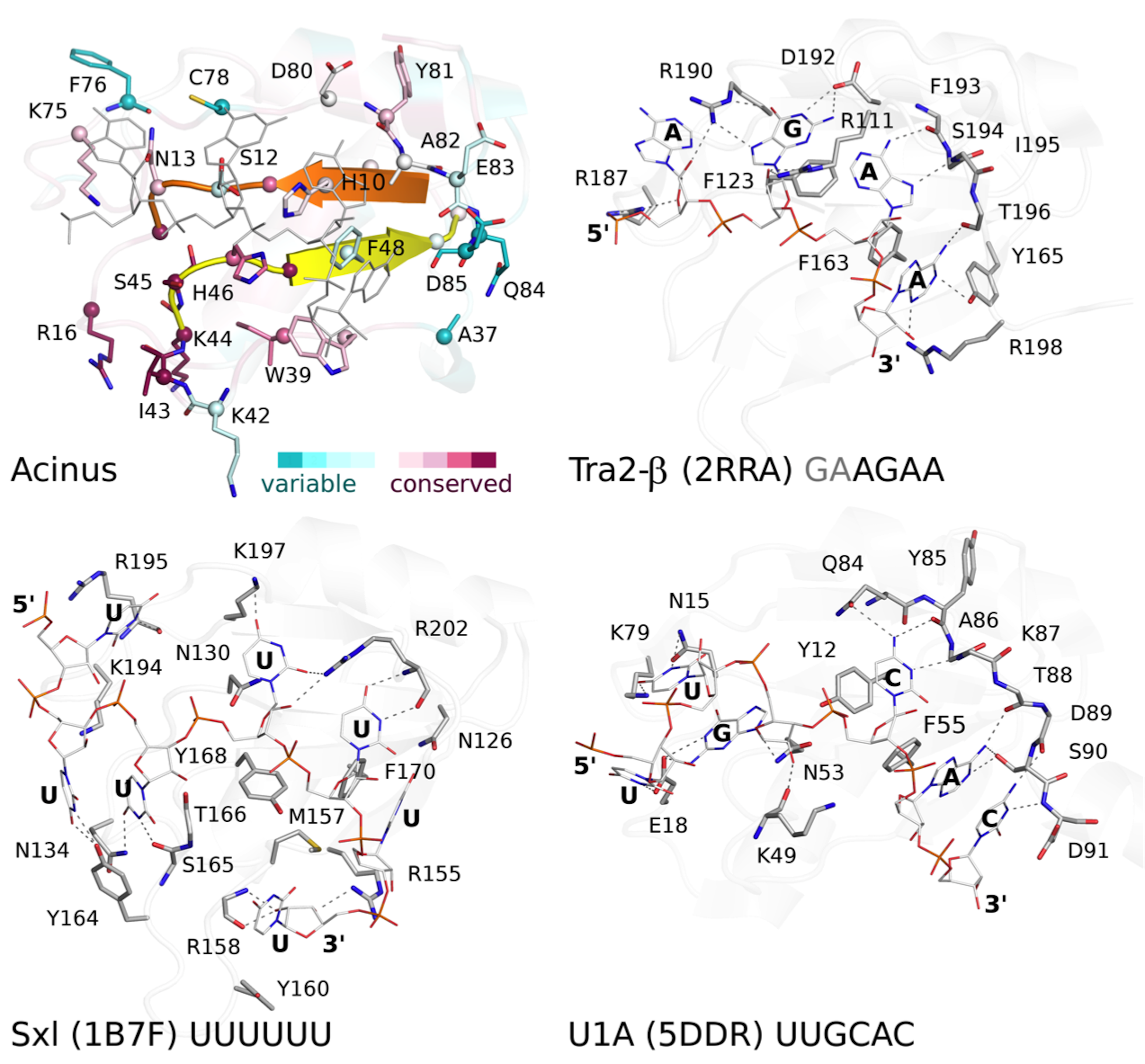

Supplement: Supplemental Information 5 — Comparison of the putative RNA binding site of AcRRM with the known RNA complexes of similar RRMs: Tra2-β, Sxl and U1A.protein-RNA (as described in Fig. 5). The protein-RNA hydrogen bonding patterns are indicated by dashed lines. [file peerj-06-5163-s005.png]
