# Supplementary material for: Crystal structure of human Acinus RNA recognition motif domain
Source: PeerJ. 2018 Jul 4;6:e5163. doi: 10.7717/peerj.5163 (PMC6057467; doi:10.7717/peerj.5163)
Supplement: Supplemental Information 4 — The UNIPROT code and amino acid range are indicated for each sequence. Multiple sequence alignment was built using MAFFT (Katoh & Standley, 2013). The homologues were collected from UNIREF90. One iteration of HMMER homolog search algorithm was used with the E-value of 0.0001. 95% maximal identity between sequences and 15% minimal identity for homologs were applied. 387 sequences that sample the list of homologues to the query were used. Bayesian method of conservation scores calculation and best fit model of substitution were used. [file peerj-06-5163-s004.pdf]

```
                      1008        1020      1030                                1040      1050 .                  1060      1070   :*   1080      1090    1098
Q9UKV3_1008_1098      GKISNIVHISNLVRPFTLGQLKELLG-RTG---------T-L-V-EE--AFWIDKIKSHCFV--------TYSTVEEAVATRTALHGVK-WPQ--SNPKFLCADYAEQDELDYHRGL
H9GCG6_903_993        GKVGNIVHICNLVRPFTLGQLKELLG-RTG---------T-L-V-EE--AFWIDKIKSHCYV--------TYSTVEEAVATRNALHGVK-WPQ--SNPKFLSADFAEQDELDFHRGL
UPI00072E54CB_1006_10 GKISNIVHISNLVRPFTLGQLKELLG-RTG---------T-L-V-EE--AFWIDKIKSHCFV--------TYSTVEEAVATRTALHGVK-WPQ--SNPKFLCADYAEQEERKLR---
UPI0006B177FE_105_195 GKISCIVHICNLVRPFTLGQLKELLG-RTG---------T-L-V-EE--AFWIDKIKSHCYV--------TYSTVEEAVATRNALHGVK-WPQ--SNPKFLSADFVEQNELDFHRGL
A0A0P7WSH2_279_369    GKITNIVHICNLVRPFTLGQLKELLN-RTG---------T-V-V-EE--GFWIDKIKSHCYV--------TYSSTEEAVATRAALHGVK-WPQ--SNPKFLSVDFSEQDELDFHRGL
F1C7H3_22_112         GKVSNIVHISNLVRPFTLGQLKELLG-RTG---------T-L-V-EE--GFWIDKIKSHCYV--------TYGSSEEAVATRAALHGVK-WPQ--SNPKVLNVDFCQQDELDFHKGL
UPI000644135A_441_530 -KVSNIIHISNLVRPFTLGQLKELLH-RTG---------T-V-L-EE--SFWIDKIKSHCFV--------TYASVEEAVATRTALHGVK-WPQ--SNPKFLSVDFSQQEELDFHRGL
A0A151NQ65_453_542    -KPSHIVHICNLVRPFTLGQLKELLS-RTG---------T-L-V-EE--GFWIDKIKSHCYV--------TYSTLEEAVLTRNSLHGVK-WPQ--SNPKFLSADFAEQDELDFHRGL
H2M9R2_962_1052       GKVSNIVHICNLVRPFTLGQLKELLS-RTG---------T-L-V-ED--GFWIDKIKSHCYV--------TYSSVEEAVATRTALHGVK-WPQ--SNPKVLSVDFCQQDELDFHKSL
UPI00085413F7_262_350 --PSCIVHVCNLVRPFTLGQLKELLS-RTG---------T-L-V-EE--NFWIDKIKSHCYV--------TYSTLEEAIATRNSLHGVK-WPQ--SNPKFLSVDFAEQDELDFHRGL
A0A1S3R3X4_1731_1821  GKVSNIVHLSNLVRPFTLGQLKELLS-RTG---------T-V-L-ED--GFWIDKIKSHCYV--------TYSSAEEAVATRAALHGVK-WPQ--SNPKFLSVDFSQQEELDFHRGL
UPI000814A1FB_1213_13 GKVSNIVHVCNLVRPFTLGQLKELLN-RTG---------T-I-V-EE--GFWIDKIKSHCYV--------TYSSAEEAQATRAALHGVK-WPQ--SNPKFLNVDFCQQDELDFHRGL
UPI0007AD4283_1397_14 GKLSNVVHVCNLVRPFTLGQLKELLN-RTG---------T-V-V-EE--GFWIDKIKSHCYV--------TYSSAEEAMATRTALHGVK-WPQ--SNPKFLSVDFCTQEELDFHRGL
H3B6Z2_916_1006       GKVTTIVHICNLVRPFTLGQLKELLN-RTG---------T-L-I-EE--TFWIDKIKSHCFA--------TYSSVEEAVATRNALHGVK-WPQ--SNPKLLTVDFAEQEQLDFHRGL
UPI00084D6AB9_1751_18 -KISCIVHICNLVRPFTLGQLKELLS-RSG---------T-I-V-EE--NFWIDKIKSHCYV--------TYSTTEEAVATRNSLHGVK-WPQ--SNPKFLSVDFAEQDELDFHRGL
UPI0006B38F76_1730_18 GKVSNIVHVCNLVRPFTLGQLKELLS-RTG---------T-L-V-ED--GFWIDKIKSHCYV--------TYSCIEEAVATRAALHGVK-WPQ--SNPKVLRVDFCEQDELDFHKGL
UPI00094EB2C0_1367_14 GKVSSIVHISNLVRPFTLGQLKELLG-RTG---------T-L-V-ED--GFWIDKIKSHCYV--------TYSSSEEAIATREALHGVK-WPQ--SNPKVLNVDFCQQDELDFHKGL
A0A1A7WMS9_756_846    GKVSNIVHICNLVRPFTLGQLKELLG-RTG---------T-L-V-DN--GFWIDKIKSHCYV--------TYSSSDEAVATRAALHGVK-WPQ--SNPKVLRVDFCEQDELDFHKGL
UPI00099F6FAC_220_310 GKVSNIVHVSNLVRPFTLSQLKELLG-RTG---------T-V-H-ED--GFWIDKIKSHCYV--------TYSSAEESIATRAALHGVK-WPQ--SNPKFLSLDFIQQEELDFHRGL
A0A1W5AKJ0_1350_1440  GNITNIIHICNLVRPFTLGQLKELLN-RTG---------A-M-L-EE--GFWIDKIKSHCYV--------TYPSTEEAVATRTALHGVK-WPQ--SNPKFLNVDFAQQDELDFHRGL
UPI0004952D10_1422_15 GKVSAIVHVCNLVRPFTLGQLKELLS-RTG---------T-L-V-EE--GFWIDKIKSHCYV--------TYSSIEEAVATRAALHGVK-WPQ--SNPKVLSVDFCQQDELDFHKGL
UPI0008F9EFBA_230_320 GKTSNIIHVYNLVRPFTLGQLKELLN-RTG---------K-L-V-EE--GFWIDKIKSHCYV--------TYSTVEEAVATREALHGVK-WPT--SNPKVLRVDFCEQDELDFHKGL
A0A1S3N950_1104_1194  GKVSNIVHVCNLVRPFTLGQLKELLN-RTG---------S-V-V-EE--GFWIDKIKSHCYV--------TYSSTEEAVATRAALHGVK-WPQ--SNPKVLTVDFCEQNELDFHRGL
UPI0009734431_1428_15 GKVSNIVHISNLVRPFTLGQLKELLS-RTG---------T-M-L-EE--GFWIDKIKSHCYV--------TYGCTEEAVATRAALHGVK-WPQ--SNPKFLSVDFSQQEELDFQRGL
UPI00097CEA79_1433_15 GKVSNIVHVCNLVRPFTLGQLKELLS-RTG---------T-L-V-ED--SFWIDKIKSHCYV--------TYSCSEEAVATRAALHGVK-WPQ--SNPKVLSVDFCQQDELDFHKGL
UPI0009B2E987_991_108 GKVSNIIHVTNLVRPFTLGQLKELLN-RTG---------S-L-L-EE--GFWIDKIKSHCFV--------TYATTEEAVATRAALHGFK-WPQ--SNPKVLGVDFCEQDELDFHKGI
UPI0007429D9E_837_927 GKISNIIHVINLVRPFTLLQLKELLN-RTG---------T-V-V-EE--GFWIDKIKSHCYV--------TYTTTEEAVATRTALHGVK-WPA--SNPKVLSVDFCEQDELDYHKGI
UPI000874C358_882_972 GKVSNIIHVTNLVRPFTLGQLKELLN-RTG---------T-M-V-ED--GFWIDKIKSHCYV--------TYATTEEAVATRAAIHGVK-WPP--SNPKVLSVDFSEQDELDFHKGV
UPI000329F967_1332_14 GKVSTIVHICNLVRPFTLGQLKELLS-RTG---------S-L-M-EE--GFWIDKIKSHCYV--------TYSSVEEAVATRAALHGVK-WPQ--SNPKVLTVDFCQQDELDFHKGL
UPI00097DD28A_873_963 GKISNIIHVTNLVRPFTLGQLKELLN-QTG---------T-V-V-EE--GFWIDKIKSHCFV--------TYATAEEAVATRTAIHGVK-WPS--SNPKVLSVDFCEQAELDFHKGI
UPI000B3DCADE_8_95    ---TNIVHLCNLVRPFTLGQLKALLG-RTG---------T-L-R-ED--GFWIDRIKSHCYV--------TYASVEEAAATREALHGVR-WPQ--SNPKVLAADFAEQEELDFHRGL
G3PLU1_930_1020       GKVSNIVHISNLVRPFTLGQLKELLS-RTG---------T-L-L-EE--GFWIDKIKSHCFV--------TYCSPEEAVATRASLHGVK-WPQ--SNPKVLRVDFCQKDELDFHKGL
M7B0L8_1113_1201      -KPSPIVHICNLVRPFTLGQLKELLG-RTG---------T-L-V-EE--AFWIDKIKSHCYV--------TYSTVEEAVLTRNALHGVK-WPQ--SNPKFLSADFAEQDEALGHPG-
Q4S0T2_201_291        GKLSNIVHVTNLVRPFTLGQLKELLN-RTG---------T-V-V-EE--GFWIDKIKSHCFV--------TYATTEEAAATREALHRVK-WPQ--SNPKVLHVDFCQQDELDFHKGI
UPI000900FFC4_1389_14 GKVSSIVHISNLVRPFTLGQLKELLS-RTG---------T-L-V-EE--GFWIDKIKSHCYV--------TYCSSDEAVATRAALHGVK-WPQ--SNPKVLSVDFCQQDELDFHKGL
UPI000BBD5ECE_1275_13 GKISNIVHVCNLVRPFTLGQLKELLS-RTG---------T-I-V-EE--DFWIDKIKSHCYV--------SYSSLEEAQATRAALHGVK-WPQ--SNPKFLSVDFCQKEELDFHRGL
UPI00077D03B4_1331_14 GKVSSIVHICNLVRPFTLGQLKELLG-RTG---------T-L-L-ND--GFWIDKIKSHCYV--------TYSSSEEAVATRAALHGVK-WPQ--SNPKVLRVDFCEQDELDFHKGL
UPI000661C41E_2269_23 GKLSNIVHVCNLVRPFTLGQLKELLN-RTG---------T-V-V-ED--GFWIDKIKSHCYV--------TYSSTEEAVATRAALHGVK-WPQ--SNPKVLSVDFCEQNELDFHKGL
H2SL09_969_1059       GKVSCIVHISNLVRPFTLGQLKELLS-RTG---------S-L-V-EE--GFWIDKIKSHCYV--------TYCSSEEAVATRAALHGVK-WPQ--SNPKVLRVDFCEQDELDFHKGL
V9KUF8_400_490        GKVSPIIHVCNLVRPFTLGQLKELLH-RTG---------S-L-L-EE--GFWIDKIKSHCYA--------TYSSVEEAVATRNSLHGVK-WPT--SNPKFLSVDFVEQDELDFHKGL
UPI000BBDEC61_1001_10 GKISNVIHVCNLVRPFTLGQLKELLN-RTG---------T-V-L-EE--GFWIDKIKSHCYV--------TYSSKEEAVATREALHGVK-WPS--SNPKVLRVDFCEQDELDFHKGL
UPI0007F93678_849_939 GKVSNIIHVTNLVRPFTLLQLKELLN-RTG---------S-V-V-EE--GFWIDKIKSHCYV--------TYATSEEAVATRTALHGVK-WPA--SNPKVLSVDFSEQDELDFHKGI
M3ZHA8_949_1039       GKVSNIIHVVNLVRPFTLLQLKELLN-RTG---------S-V-V-EE--GFWIDKIKSHCYV--------TYTTTEEAVATRTALHGVK-WPP--SNPKVLGVDFCEQDELDYHKGI
UPI000A1C4716_206_296 GKVSKIIHVTNLVRPFTVGQLKELLS-QTG---------S-V-V-ED--GFWIDKIKSHCYV--------TYSTEEEAVSTRNALHGVK-WPQ--SNPKVLSVDFSEQDELDFHKGI
UPI0009B495BE_1341_14 GKVSNIVHICNLVRPFTLGQLKELLS-RTG---------I-L-V-EE--GFWIDKIKSHCYV--------TYSSSEEAVATRAALHGVK-WPQ--SNPKVLSVDFCQQDELEFHKNL
UPI0007B92203_913_100 GKTSHIIHVCNLVRPFTLGQLKELLN-RTG---------T-L-V-EE--GFWIDKIKSHCYV--------TYSTIEEAVATREALHGVK-WPT--SNPKVLRVDFCEQYELDFHKGL
UPI000496A047_876_966 GKISNIIHVTNLVRPFTLGQLKELLN-RTG---------S-V-V-EE--GFWIDKIKSHCYV--------TYATSEEAVATRTALHGVK-WPP--SNPKVLSVDFCEQHELNFHKGV
UPI0002A4A609_812_902 GKVSNIVHVTNLVRPFTLLQLKELLN-RTG---------T-M-V-EE--GFWIDKIKSHCYV--------TYSATEEAVATREALHGVK-WPP--SNPKVLRVDFCEQDELDFHKGV
UPI0009B34807_858_946 GKVSNIIHVTNLVRPFTLGQLKELLS-RTG---------S-V-V-EE--GFWIDKIKSHCYV--------TYATTEEAVATRTALHGVK-WPQ--SNPKVLNVDFCEQHELNFHK--
UPI000B8F92B5_912_100 GKVSNIIHVTNLVRPFTLGQLKELLN-QTG---------S-V-V-EE--GFWIDKIKSHCYV--------TYATSEEAIATRAALHGVK-WPT--SNPKVLNVDFCEQDELDFHKGV
UPI0009048405_842_932 GKVSNIIHVTNLVRPFTLGQLKELLN-RTG---------S-V-V-EE--GFWIDKIKSHCYV--------TYATTDEAVATRAALHGVK-WPT--SNPKVLSVDFCEQTELDFHKGV
UPI000814B190_958_104 GKVSNIIHICNLVRPFTLGQLKELLN-RTG---------T-V-V-EE--SFWIDKIKSHCYV--------TYSSKEEAVATCEALHGVK-WPS--SNPKVLRVDFCEQDEVDFHKGL
UPI00094EC2C1_747_837 GKLSNIVHVTNLVRPFTVGQLKELLK-QTG---------S-M-V-ED--GFWIDKIKSHCYV--------TYTTTEEAAATRESLHGVK-WPQ--SNPKVLSVDFCEQDELDFHKGL
F1Q511_909_999        GKTSNIIHVLHLVRPFTLGQLKLLLN-RTG---------T-M-V-EE--GFWIDKIKSHCYV--------TYSTVEEAVATREALHGVK-WPA--SNPKVLRVDFSDQDELDFHKGL
UPI0006B3A046_497_587 GKVSNIIHVTNLVRPFTLLQLKELLN-RTG---------S-V-V-EE--GFWIDKIKSHCYV--------TYTTAEEAVSTRTALHGVK-WPA--SNPKVLNVDFCEHDELDFHKGI
UPI000554C5C1_263_353 GKVSTIIHVTNLVRPFTLGQLKELLN-RTG---------S-V-L-ED--CFWIDKIKSHCFV--------TYGTTEEAVATRAALHGVK-WPS--SNPKVLNVDFCEQDEMDIHKGI
UPI0007DC9524_1329_14 GKVSNIVHVCNLVRPFTLGQLKELLS-RTG---------T-L-V-DD--GFWIDKIKSHCYV--------TYSSSEEAVATRAALHGVK-WPQ--SNPKLLSVDFCLQEELDFHK--
H2TAD9_889_979        GKLSNIIHVTNLVRPFTLGQLKELLN-RTG---------T-M-V-EE--GFWIDKIKSHCIV--------TYATTEEAVATRDALHRVK-WPL--SNPKVLSVDFCQQEELDFHKGI
```

```
                      1008           1020      1030                     1040      1050 .                1060      1070    :*    1080      1090   1098
UPI00072EE877_1255_13 GQVSSIVHIWNLVRPFTLGQLKELLS-RTG---------T-L-V-ED--GFWIDKIKSHCYV--------TYSSAEEAVATRAALHGVK-WPQ--SNPKVLRVDFCQQDELDYQK--
UPI0007429473_1298_13 GQASNIVHIWNLVRPFTLGQLKELLS-RTG---------T-L-V-ED--GFWINKIKSHCYV--------TYSTIEEAVATQAALHGVK-WPQ--SNPKFLRVDFCQQEELDHQR--
UPI000496F436_263_353 GQISKIIHVTNLVRPFTLGQLKELLN-RTG---------S-V-L-ED--GFWIDKIKSHCYV--------TYATTEEAVSTRNAIHGVK-WPS--SNPKVLSVDFCGQDELDFHRGI
A0A1A8E557_207_297    GKVSNIIHVTNLVRPFTLLQLKELLN-RTG---------S-V-V-EE--GFWIDKIKSHCYV--------TYASTEEAVSTRTALHGVK-WPA--SNPKVLSVDFCKQAELDFHKGI
UPI00064418ED_927_101 GKVSKIIHVRNLVRPFTLGQLKELLS-RTG---------A-V-V-EE--GFWIDKIKSHCYV--------TYSSTEEAVATRTALHGMK-WPL--SNPKVLNVDFSEQEELDFQKGL
UPI000802B86F_967_105 GKTSNIIHVCNLVRPFTLGQLKELLN-RTG---------A-V-V-ED--GFWIDKIKSHCYV--------TYASTEEAVATREALHGVK-WPS--SNPKVLRVDFCEQKELDFHKGL
A0A0F8C817_928_1017   -KISNIIHVTNLVRPFTLGQLKELLK-RTG---------V-V-M-EE--GFWIDKIKSHCFV--------TYTTTKEAFASRAALHGVK-WPQ--SNPKVLSVEFCEQAELDFHKGV
UPI0006B7CC38_120_203 -------SPSGPVRPFTLGQLKELLG-RTG---------R-L-K-DD--GFWIDRIKSHCFV--------TYCSVEEAVATREALHGVK-WPQ--SNPKVLAADFAQQEELDFHRGL
UPI0003F0DCE1_713_801 --VTKIVHVMNLVRPFTINQLKELLS-RSG---------T-I-V-EE--GFWIDKIKSHCFV--------TYSTEEEAIKTRDALHGTK-WPS--SNPKNLIVEYAVQEEIDRHKGV
UPI000948218F_837_925 --VSQIVHILHLVRPFTLGQLKELLG-RTG---------T-I-V-EDK-GFWIDKIKSHCFV--------KYESEEEAVKTRQALHGVR-WPS--SNPKTLLVDFADQDELDIHLG-
A0A0L8GBT8_657_744    --VSKIVHVRNLVRPFTIGQLKELLK-RTG---------A-V-V-EN--GFWIDKIKSHCFA--------KYETEEQAILTRKALHGTK-WPS--SNPKILRVEYATDDEVSYYRS-
UPI000719AFA5_609_695 ---STLVHIRNLVRPFTLNQLKELLG-RSG---------K-L-V-DS--SFWIDKIKSHCFA--------EFETVDEAMALRADLHGVK-WPS--SNPKVIRVDFTEPAELDFHRE-
UPI000BAF2231_796_880 --VSNIIHIRNLVRPFTLNQLKELLK-RTG---------N-L-V-DD--GFWIDNIKSHCIV--------TYENEEGAKATRAALHGTK-WPQ--SNPKILRVEYATKDELDR----
K1QEG1_472_556        --VSNIIHIRNLTRPFTLNQLKELLK-RTG---------S-L-V-EE--SFWIDNIKSHCIV--------TYENEEQATATRASLHGTT-WPQ--SNPKILKVEYASKDELER----
A0A1S3J0R6_705_791    --VSKIVHVTGLVRPYTLGQLKTLLG-QTG---------T-L-V-ED--GFWIDNIKSHCYA--------TFETEEQAKETRQALHGTR-WPL--SNPKILRVDFATEDELSFHK--
T1JN81_147_234        --VNRIVHIRNLVRPFTINQLKELLG-RTG---------T-L-V-DG--GFWTDKIKSKCYA--------TYETQEQAMKTRQTLHGTQ-WPP--SNPKNLIVDFADDDELQFHMS-
UPI0009E51C95_603_684 ---SEVLHVKNLVRPFTLNQLKDLLG-KHG---------T-L-V-EG--GFWIDKIKSHCYV--------VYSDVEEAAACRRALHGIK-WPV--TSPKILDVDFADQDEM------
A0A210PFD3_828_914    --VSKIIHVRNLVRPFTLNQLKDLLR-RTG---------N-I-V-EN--EFWIDKIKSHCFV--------TYETEENAVQTRKALHNTK-WPS--SNPKLLWLEFASPEEIEFNK--
UPI00077FA035_1613_17 GPISKIVFIQNLVRPFTLLQLKDLLR-QTG---------N-F-I-ED--DFWIDKIKSKCFV--------TYTTEEEAIKTRNALNGVR-WPS--SNPKILSAEFSTEEEFEHHR--
UPI000B90D3B4_803_890 ---SAVVHVTNLVRPYTLGQLKELLC-RSG---------Q-LEA-ED--GFWIDNIKSHCFA--------VFTSVEEAVACRDSLHGTR-WPS--SNPKMLRVDFASKEEMDRHTG-
T1KU57_15_103         -KTSNIIFIRNLVRPFTLVRLKELLE-KNG---------K-L-V-ED--QFWIDKIKSKCYA--------TYEKEEDAINTREALHGIS-WPD--ANPKKLIIDFATKEELEYHLN-
A0A067RGG4_866_953    -KASNVLFISNLVRPFTVNQLKELLA-RTG---------N-I-V-EG--GFWIDKIKSKCYV--------QYETETEARETRHALHGVR-WPV--SNPKQLCVDFGKKEDMDLAQ--
UPI0006B0997C_1566_16 ---SNIISIRNLVRPFTLLQLKDLLA-KTG---------T-L-V-DG--GFWIDKIKSKCCV--------TYETEEQAEETRKALHGVQ-WPI--SNPKTLIVDFATKEELDQH---
F6LVT0_57_143         ---SKILHVMNLVRPFTIPQLKALLG-RFG---------T-L-A-EE--GFWINTIKSHCYA--------TFHSVEGAEKCRESLHGAT-WPQ--SNPKTLNIEFATQSELDRYLG-
A0A087UBF0_986_1071   ---SKIIFVQNLVRPFTLLQLKELLK-QSG---------N-L-I-EE--HFWIDKIKSKCFV--------MYETEEEAIKARNALNGTR-WPV--SNPKILSVEFATEDDIEYHK--
UPI000B98F975_607_688 ---SDVLFITNLVRPFTIPQLKNLLA-RTG---------K-I-A-EN--GFWIDKIKSKCYV--------QYSDVEEAIETRHALHGVR-WPV--SNPRTLCVEFANHDDM------
A0A0A1WG15_359_443    -KTSNVLFITNLVRPFTVLQLKGLLA-RTG---------K-L-V-ED--GFWIDRIKSKCYV--------QYETEDEAIETRHALHGVR-WPV--SNPKCLIVDFAKRADMD-----
A0A088A2F5_203_290    -KPTNILLIKNLVRPFTLNQIKELLS-RTG---------T-I-V-EN--GFWMDRIKSKCFV--------EYANEDQAFETRQALHGIS-WPV--SNPKRLHVEYATKDDMELAR--
UPI0008116533_356_440 -KPSNVLFITNLVRPFTVLQLKGLLA-RTG---------K-M-V-EE--GFWIDRIKSKCYV--------QYETEDEAVETRHALHGVR-WPV--SNPKCLIVEFARKSDLD-----
W4XHR7_854_941        --TSNIVHVINLVRPFTLPQLKELLG-RYG---------T-L-S-AD--GFWINNIKSHCYA--------TFESSDGAVACRDALHGTT-WPS--SNPKTLRVEFADLNELQRHMG-
UPI000A2A9C61_664_741 ------IHVNNLVRPFTLNQLKDLLG-KHG---------T-L-V-DD--GFWIDKIKSHCYA--------VYSDIDGAKATRAALHGVK-WPP--TSPKILSVEFEDDHQ-------
R7VLC9_312_398        --VTKLVHIRHLVRPYTLNQLKELLS-QHG---------T-L-A-E---GFWIDKIKSHCIA--------SYETEEAATAARAALHGSK-WPS--SNPNALKVDYATVEELQFHLG-
UPI000B926133_520_603 ---SNILYITNLVRPFTLPQLKNLLQ-RTG---------R-I-I-EN--GFWIDKIKSKCFV--------IYENEEQAVETRHALHGVT-WPV--SNPKSLQVDFSTQEDFDK----
UPI0005CEF3FA_683_766 --ATNILLIRNLVRPFTLNQIKELLS-RTG---------T-I-V-EN--GFWMDRIKSKCFV--------EYANEDQAFETRQALHGVS-WPL--SNPKKLIVDYATKEDMN-----
E0VVJ6_632_711        -KPSNILYIVNLVRPFTIPQLRELLA-RTG---------T-I-V-ED--GFWIDKIKSKCYV--------KYETEEQAKTTRHALHGVR-WPV--SNPKQLNVDFGT----------
A0A182H862_457_537    ---SNILYITNLVRPFTVLQLKSLLA-RTG---------K-I-V-EN--GFWIDKIKSKCFI--------KYETEDEAVETRHALHGVR-WPA--SNPKCLHVDFGTESA-------
UPI0005ABD393_649_733 --TSNILLIKNLVRPFTLNQIKELLS-RTG---------T-I-V-EN--GFWMDRIKSKCFV--------EYANEDQAFETRQALHGIS-WPT--SNPKKLQVEYSVKEEMDK----
K7IQ96_683_766        ---TNVLLIKNLVRPFTLHQIKELLS-RTG---------T-I-V-EN--GFWIDRIKSKCFV--------EYTNEDQAFETRQALHGVS-WPL--SNPKKLIVEYATKEDMEA----
U5EU16_428_510        ---CNILYITNLVRPFTVLQLKGLLA-RTG---------K-I-V-EN--GFWIDKIKSKCYV--------KYETEDEAVETRHALHGVR-WPT--SNPKCLNVDFGASDDME-----
A0A0N8AW84_345_427    ---SSVLNVSNLVRPFTINQLKELLA-RTG---------H-L-V-EG--KFWIDRVKSSCLV--------QYATEDEAEETRAALHGIH-WPT--SNPKTLLVDYSTVEDLE-----
UPI000811588E_419_501 ---STVLFISGLVRPYTLPQLKKLLC-LNG---------L-I-N-EE--KFWIDKIKSKCYA--------VYSTVEEAVKTREALHGLK-WPQ--SSPKDLCVEFATLEDID-----
UPI0006C990DD_685_768 --ATNILLIRNLVRPFTLNQIKELLA-RTG---------T-I-V-EN--GFWMDRIKSKCFV--------QYANEDQAFETRQALHGVS-WPV--SNPKKLIVEYATKEDME-----
A0A1S4H0J7_601_683    ---SNILYITNLVRPFTVLQLKGLLA-RTG---------K-I-V-EN--GFWIDKIKSKCYV--------KYETEDEATETRHALHGIR-WPV--SNPKCLVVDFGSEEAMD-----
A0A1J1I2N1_466_549    --SSNILYITNLVRPFTLLQLKHLLL-RTG---------K-I-V-EN--GFWIDKIKSKCYV--------KYETEDEASETRHALHGVT-WPS--SNPKCLNVDFGKEEDME-----
A0A194R348_632_718    -KQSNILYITNLVRPFTLSQLRNLLQ-RTG---------R-I-T-EN--GFWIDRIKSKCFV--------IYENEDQAVETRHALHGVT-WPV--SNPKTLHVDFSTQEDFDHA---
A0A158NJU1_618_703    -KPTNILLIKNLVRPFTLNQIKELLS-RTG---------T-I-V-EN--GFWMDRIKSKCIV--------EYSNEDQAFETRQALHGIS-WPM--SNPKKLHVEYATKEDMET----
A0A0B7AQS8_916_995    ----RVVRITGLVRPFTVGQLKELLK-RTG---------E-L-D-DE--YFWINAIKSHCLA--------AYKTQEEASATRAALHGTR-WPQ--SNPKILIVDFATVED-------
E9HGW7_916_998        ---SSVLNVSNLVRPFTINQLKELLA-RTG---------H-L-I-DG--KFWIDRVKSSCLI--------QYETEEEAEETRAALHGIH-WPT--SNPKTLLVDYSTVEELE-----
UPI0006253782_644_731 -KPTNVLLIKNLVRPFTLNQIKELLS-RTG---------T-I-V-EN--GFWIDGIKSKCYV--------VYSNEDQAFETRQALHGIS-WPV--SNPKRLQVEYGTKEDMELAR--
UPI0006C9B195_635_718 --ATNILLIKNLVRPFTLNQIKELLS-RTG---------T-I-V-EN--GFWMDRIKSKCYV--------EYKNEDQAFETRQALHGIS-WPL--SNPKKLIVEYANKEDME-----
A0A1B6C4K4_157_239    ---SSVLYITNLVRPFTVQQLKNLLL-RTG---------T-I-A-EN--GFWIDSIKSKCYV--------EYTTEDAAVETRHALHGVR-WPV--SNPKTLIVDFSTHDDMA-----
UPI0006CEDBF5_581_665 -KATNILYITNLVRPFTVPQLKELLA-RTG---------T-L-A-PN--GFFIDKIKSKCYV--------KYTTVEMAVETRHALHGVR-WPV--NNPKTLKVEFSNEQNLN-----
UPI0004CDCB05_721_804 ----SVLLIKNLVRPFTLNQIKELLS-RTG---------S-I-V-EN--GFWMDRIKSKCFV--------EYSNEDQAFETRQALHGIS-WPL--SNPKKLQVEYATKEEMEQA---
UPI0007E8658D_460_544 --ASHVLYITNLVRPFTVLQLKGLLA-RTG---------K-I-VDED--GFWIDRIKSKCFV--------AYSTEDEAIETRHALHGVR-WPV--SNPKCLNVDFGSRTDMD-----
A0A1B6MCH3_322_405    ---TSVLYITHLVRPFTVQQLKNLLL-RTG---------T-I-T-DN--GFWIDKIKSKCYV--------EYTTEDAAVETRHALHGVR-WPT--SNPKTLVVEFASHDDLTI----
UPI000771E011_602_684 ----SILLIKNLVRPFTLNQIKELLS-RTG---------T-I-I-EN--GFWMDRIKSKCFV--------EYANEDQAFETRQALHGIS-WPV--SNPKRLHVEYANKEDMEA----
A0A1E1WU44_526_610    -KQSSILYITNLVRPFTLPQLKNLLQ-RTG---------K-I-V-EN--GFWIDRIKSKCFV--------QYDNEDQAVETRHALHGVT-WPV--SNPKTLQVDFSSEEAFE-----
A0A1Q3F462_462_540    ---SSILYITNLVRPFTVLQLKSLLA-RTG---------K-I-V-EN--GFWIDKIKSKCFV--------KYDNEDEAIETRHALHGVR-WPT--SNPKCLHVDFGTE---------
```

```
                      1008        1020      1030                     1040        1050 .               1060      1070  :*    1080      1090   1098
UPI00076FD3CB_637_723 -KATNVLLIKNLVRPFTLNQIKELLS-RTG---------T-I-I-EN--GFWIDGIKSKCYV--------VYANEDQAFETRQALHGIS-WPL--SNPKRLQVEYATIEDVEKA---
A0A1W4WJU4_556_641    --VSAVLFITNLVRPFTLKQLKDLLE-RTG---------K-I-K-QN--GFWTDKVKSKCYV--------HYETEEEAEATRNALHGVN-WPV--GNAKQLCIEYATEEDLENA---
UPI000359CFF7_879_964 --VDRVIRIACLVRPFTVNQLKELLR-RSG---------E-L-D-EE--YFWINDIKSHCLA--------AYKTEEDAVKARSCLHGTK-WPQ--SNPKILVVDYATLEDVEHR---
UPI000B39EC5F_555_639 -KQSCILYITNLVRPFTLPQLKNLLQ-RTG---------R-I-T-EN--GFWIDRIKSKCFV--------QYETEDQAVETRHALHGVT-WPV--SNPKTLQVDFSTQEAFD-----
A0A1A9W021_452_531    --TSSVLYITNLVRPFTVLQLKGLLA-RTG---------K-I-V-ED--GFWIDRIKSKCYV--------EYETEDEAIETRHALHGVR-WPV--SNPKCLNVDFGLK---------
A0A1L8DXC1_435_515    ---SCILYITNLVRPFTVLQLKGLLA-RTG---------R-I-V-EN--GFWIDKIKSKCYV--------QYETEDQAVETRHALHGVR-WPT--SNPKCLHVDFGSEGD-------
A0A1Y3AS26_327_409    ---STVLFITGLVRPYTLMQLKKLLN-SNG---------Q-I-D-EE--KFWIDKIKSKCYA--------TYSSVEEAVKTRKNLHNLK-WPQ--SSPKNLCVEFATLEDID-----
D6WKZ2_593_676        --VSNILYITNLVRPFTVKQLKELLE-RTG---------K-V-Q-ED--GFWTDRIKSKCYV--------QYETQEEAEATRNALHGVQ-WPI--GNGKKLIIDFATAEDME-----
UPI0005D0BA8C_536_620 -KQSHILYITNLVRPFTLPQLKNLLQ-RTG---------R-I-A-EG--GFWIDRIKSRCFV--------IYENEDQAVETRHALHGVT-WPV--SNPKTLQVDFSSQEAFD-----
UPI0008737D84_539_622 ---SDVLFITNLVRPFTVKQLKELLE-RTG---------K-I-K-EE--GFWTDRIKSKCYV--------QYETSEEAEATRNALHGVH-WPI--GNGKKLVIDYATAEDLDK----
A0A0L7KY09_582_666    -KQSSVLYITNLVRPFTLPQLKNLLQ-RTG---------R-L-V-EN--GFWIDKIKSKCYV--------IYENEDQAIETRHALHGVT-WPV--SNPKALQVDFSTLEAFD-----
A0A1B6JFI6_610_692    ---TSVLYITHLVRPFTVQQLKNLLL-RTG---------T-I-A-EN--GFWIDKIKSKCYV--------EFTTEDAAVETRHALHGVR-WPP--SNPKTLVVEFASHDDLI-----
A0A212F643_660_743    -KQSNILYITNLVRPFTILQLKNLLQ-RTG---------R-I-M-EN--GFWMDKIKSKCFV--------KYENEDQAIETRHALHGVT-WPV--SNPKTLHVDFSTEEDF------
T1FJ08_841_919        ----KFVHVFNLVRPFTVGQLKELMG-KYG---------K-V-I-EE--GFWIDKIKSHCI----------YETIDQSTNCRQSLHGRK-WPA--SNPKLLRVDYCDEEEV------
E9JEG5_601_685        -KQSNILFITNLVRPFTLLQLKNLLQ-RTG---------R-I-E-EN--GFWIDKIKSKCYV--------KYETEDQAVETRHALHGVT-WPV--SNPKTLHVDFSTTEAFD-----
T1PGR8_450_530        ---SCVLYITNLVRPFTVLQLKGLLA-RTG---------K-I-V-EN--GFWIDRIKSKCYV--------EYETEDEAIETRHALHGVR-WPA--SNPKCLNVDFGNKSD-------
UPI0002B464C9_403_482 --VSCYIHITGLVRPFTVNQLKVLIG-RTG---------T-I-S-EN--GFWINQVKSHCYV--------QLSSEEQAAATRNALHGLK-WPS--GTPKVLKVDFALN---------
B4LS17_442_526        --ASHVLFITNLVRPFTVLQLKGLLA-RTG---------K-I-IEED--GFWIDRIKSKCYV--------AYATEDEAIETRHALHGVR-WPV--SNPKCLNVDFGSRTDMD-----
B3MNR0_437_521        --ASHVLYITNLVRPFTVMQLKGLLA-RTG---------K-I-VEDD--GFWIDRIKSKCYV--------AYSSEDEAIETRHALHGVR-WPV--SNPKCLNVDFGSRLDMD-----
A0A1B6F8L1_3_80       --------IRNLVRPFTVKQLKDLLS-RTG---------V-I-V-DN--GFWTDRIKSMCFV--------CYETKEQAELSRNSLHGVK-WPI--GNRKKLCAEFSTESEME-----
B3RVV3_278_366        --VNEIVHITNLVRPFTLVQLKEILR-EYG---------T-I-K-ED--EFWIDNIRSRCMV--------KYSSTEEATSARNNLYGKR-WPS--TSPKRLTVEFSNQEEMDRARGI
UPI00083BE52F_711_793 ---SEVLFITNLVRPFTLKQLKELLE-RTG---------T-I-K-ED--GFWTDRIKSKCYV--------HYQTVEEAENTRNALHGVH-WPI--GNGKKLIIDYSTPENLN-----
A0A0L0BQN0_480_560    --ASSVLFITNLVRPFTVLQLKGLLA-RTG---------K-I-V-EN--GFWIDRIKSKCYV--------EYETEDQAIETRHALHGVR-WPA--SNPKCLNVDFGIKN--------
UPI00094E7D49_582_665 --VSEVLFITNLVRPFTVKQLKELLE-RTG---------K-L-K-ED--GFWTDKIKSKCFA--------CYETAEEAEVTRNALHGVH-WPI--GNGKKLIIDYSTMEDME-----
A0A0A9YFX2_706_788    -KASCILFITNLVRPFTIGQLKELLN-RTG---------T-I-S-PN--GFYIDKIKSKCFV--------KYNDVEMAIETRHALHGVR-WPV--TNPRTLRVEFSTDEA-------
UPI00067D2E68_532_614 ---SCILFISNLVRPFTLPQLKNLLQ-RTG---------R-I-E-EN--GFWIDRIKSKCFV--------KYVSEDQAVETRHALHGVT-WPV--SNPKTLQVDFSTQEAFD-----
T1GPJ7_98_178         ---SSILYITNLVRPFTIMQLKGLLA-RTG---------K-I-V-DN--GFWIDRIKSKCYV--------KFENEDEAIETRHALHGVR-WPT--SNPKCLNVEFGTEEA-------
A0A1Y1KVX1_619_704    --ISELLYITNLVRPFTLKQLKELLE-RTG---------K-I-R-EN--GFWTDRIKSKCYA--------YYETAEEAEATRNALHGVH-WPI--GNGKKLIIEYSTEEDLEKA---
UPI0006744EC2_869_949 ----RVIRISGLVRPFTIGQLKELLK-RSG---------E-L-D-ED--NFWINDIKSHCLV--------AYKCQEDAVKARAALHKTR-WPQ--SNPKILLVDFATIKDV------
A0A183IA40_40_127     --VSNIIHVRCLTRPFTTRSLFELLG-QFG---------A-F-S-KE--EFWIDGIKSHCLV--------KYDSEESALHARNALHNLQ-WPP--SNQKVLRVDFATQDDLDRHCG-
A0A0N7ZAG7_3140_3221  ---SRVVYIQNLVRPFTINQLRELLQ-RTG---------R-L-T-ES--GFWIDNIKSKCLA--------MYETEDQALETRMALHGVR-WPV--SNPKNLFVDFSTETEM------
A0A023G7N3_185_270    ---SRVLFVRNLVRPFTLNQLKQLLQ-EFG---------D-T-V-DS--EFWIDKIKSKCFV--------TYATEEDAVKAREALHNLR-WPL--CNPKILHVDFSSPEEMIRHK--
A0A183IV79_285_373    --VSNIIHVRCLTRPFTTRSLHQLLA-QFG---------T-F-S-KD--DFWMDGIKSHCLV--------KYENEEDALRARNALHNLQ-WPP--SNQKVLRVDFATQDDLDRHRGL
A0A1D2N0N0_217_300    ---SSVLLITNLVRPFTLPQLKEMLQ-RTG---------N-L-V-----DIWVDKIKSKCIA--------KFDSQEEAFETRAALHGVC-WPS--SNPKSLHVDYTTLDNLDDHK--
A0A0T6AWE5_554_636    ---SSVLFITNLVRPFTLKQLKELLE-RTG---------K-I-K-EN--CFWTDRIKSKCYV--------QYENVLDAEATRNALHGVH-WPI--GNGKKLVIDYGTEDDLE-----
A0A132AES6_397_479    ---STVLFITGLVRPYTLQQLKKLLN-DHG---------K-I-D-EE--KFWIDPIKSKCYV--------VYSTIDEAIETRKALHNLK-WPQ--SSPKNLHVEFATLEDID-----
G5DYZ8_175_230        ----------------------------------------------S--NFWIDKI-SHCYV--------TYSTTEEAVATRNSLHGVK-WPQ--SN-KFLSVDYAEQDELDFHRGL
A0A131XFN3_315_398    ---SRILFVRNLVRPFTLNQLKQLLQ-EFG---------D-T-V-DS--EFWIDKIKSKCFV--------TYVSEEDAIKAREALHNLR-WPL--CNPKILHVDFSSPEEMER----
UPI0006D501CF_641_721 ----EVLFITNLVRPFTIPQLRELLA-RTG---------T-I-A-SN--GFYIDKIKSKCYV--------KYTDIEMAVETRHALNGVR-WPV--NNPKMLKVEFATPEDM------
A0A023F7S4_649_731    -KATEVLFITNLVRPFTVPQLRELLA-RTG---------T-I-A-PN--GFYIDKIKSKCYV--------KYTDLEMAIETRHALHGVR-WPV--NNPKTLRVEFALPED-------
B7PGW6_38_116         ------LFVRNLVRPFTLNQLKQLLL-EFG---------E-T-V-DS--EFWIDKIKSKCFV--------TYTTEEEAAKAREALHNLR-WPL--CNPKILHVDFSTPEEM------
A0A177AVD3_211_293    -----YLHISNLRRPFLVAQLKNFLR-QTG---------N-Y-D-PE--SFWIDDIKTHCFV--------KYDTIEEAEETRFALHGLI-WPI--SNRVTLIADFASEEEYLSH---
UPI00096B250C_626_709 ---SEVLYISNLVRPFTLKALKELLE-RTG---------K-I-VEEK--GFWTDKIKSKCIV--------RYEKQEDAEATRNALHGVH-WPI--GNGKKLIIEYSTVEELE-----
A0A1D1V5H4_532_613    -----LLHIKNLTRPFTEKQLKEMLS-KTG---------T-L-V-EGPDHFWLDSIKSQCIA--------TFSTVEEAEATRRALFNLV-WPA--SNPRQLFAEFIREDEF------
A0A1W2W741_650_735    ---SCVLHVSNLVRPFAITQLKQLLA-ENG---------P-F-K-ED--SFWIDTIKSHCFV--------IYEDKETAEITRKCLHRLQ-WPV--SSPKNLQVEFSNEKEIEWHK--
A0A226EZK2_614_693    ---SEIILVSNLTRPFTLPQLRELLQ-RTG---------T-L----E--DLWVDKIKSKCIA--------KYEKQEDAFETRAALHGVN-WPP--SNPKALQVDYTTEETL------
A0A1S3DGW8_41_119     -----VLYIYNLTRPFTLGQLRELLS-RTG---------K-I-V-PD--GFWINAIKSKCYV--------EYENEEQAEETRHALHEVR-WPV--TNDKVLKVEFTTKDA-------
A0A1Z5LD87_26_105     -----TLFVRNLVRPFTLNQLKQLLK-EFG---------D-I-D-ES--EFWIDKIKSKCFV--------TYLSEDDAVKARQALHQLR-WPL--CNPKILHVDFSTPEEM------
A0A146UKZ1_185_266    --VSDTLHIRGLVRPFTLSQLKAILN-RHA---------P-M-V-EP--DFWIDSIKSHCFV--------RYRSTQEANLVREELHRLR-WPS--SSPQVLKVDFALKDE-------
UPI0008F9B8DE_502_583 ---SSVLLITNLVRPFTIIQLRELLQ-RTG---------K-I-Q-EG--GFWIDAIKSKCIV--------EFENEDQAVETRHALHDVR-WPV--SNPKLLHVDFCKKEDL------
A0A1D6IKB0_498_574    ------LRIDRFVRPFTLKAVKELLG-KTG---------S-I----C--SFWMDEIKTHCYV--------TYSSVEEAVATRNAVYDLQ-WPS--NNNNYLIAEFVDPEEV------
A0A1W0WBS5_559_642    --VTRMIYIRNLVRPFSLPMLKELLQ-KTG---------T-L-V-DDAEHFWIDGIKSQCIA--------TYSTEEEAEASRKALFGLT-WPA--TNPQKLTVEFIREED-------
UPI0007636AA3_568_650 ---TSVIRITNLVRPFTVGQLRDLLQ-RTG---------K-I-V-PN--GFWIDTIKSQCIV--------EYENEDQAIETVYALNDSQ-WPS--TNPKFLRVVFTSKEELN-----
A0A0A9J3G2_208_284    ------LRIDGFVRPFTLKTVQELLG-KTG---------S-V----C--SFWMDHIKTHCYV--------TYSSVEEAVATRNAVYNLQ-WPP--NNHSYLVAEFVDPQEV------
K3ZR26_556_633        ------LRIDRFVRPFTLKAVQELIG-KTG---------S-V----C--SFWMDHIKTHCYV--------TYSSVEEAVATRNAIYNLQ-WPP--NNRNYLVAEFVDPEEVE-----
A0A0B2UR38_40_124     ---NQVLMIRQLTRPFTSKQLQQMLS-TFG---------T-I-V-EG--GFWIDNIKSTCIV--------KYSSVEEAIVARGRLHNVV-WPP--HSPKALKVDYSDDEGLARH---
UPI000B5D3632_497_576 ---TNSLRIDNFVRPFTLKAVQELLG-KTG---------K-V----T--SFWMDQIKTHCYV--------SYSSVEEAVETRNAVYNLQ-WPP--NGGRFLAAEFVDPKEV------
E4WYW0_360_444        GEESQVVHVLNLVRPFTNKQLLEFLK-EAG---------E-L-D-ED--SFWIDRIKSNCIV--------KYNALESAINCRKLVHGKN-WPS--SNPKELRVMYSTEEKL------
```

```
                    1008       1020      1030                     1040       1050  .            1060       1070    :*   1080       1090     1098
A0A218XBD4_529_608     ---TNSLRIDRFLRPFTLKAVQELLG-KTG----------T-V----K--SFWMDQIKTHCYV---------TYSSVEEAVETRNAVYNLQ-WPP--NGGRLLVAEFVEPEEV------
A0A1X7V4K9_702_782     ---SRILHVENLKRPFTLLQLKDFLQ-EDG----------P-M-I-EG--GFWTNKIKSHCIA---------IFTSTETAVAARARMHGLK-WPS--INPQHLKVDFLSNQE-------
UPI00090CEDDE_538_616  ----NSLRIDRFLRPFTLKAVQELLG-KTG----------N-V----S--SFWMDQIKTHCYV---------TYSSTEEAIETRNAVYNLQ-WPP--NGGRFLLAEYVEPEEV------
D5ACP8_24_99           -------KIERFLRPFTIKALKELLS-ETG----------T-I----R--YFWIDQIKTHCYV---------TYSSVEEATATRNALYNLK-WPI--IGGRLLIAEFVDPEEV------
UPI00057AAF58_462_541  ------LRIDRFLRPFTLKAVQELLA-KTG----------T-V----C--SFWMDQIKTHCYV---------TYSSVEEAVATRNAVYNLQ-WPT--NGGKLLLAEFVDPQEVKHH---
UPI0004E5A783_456_535  ------LRIDHFLRPFTLKAVQELLA-KTG----------T-V----C--SFWMDQIKTHCYV---------TYSSVEEAVATRNAVYNLQ-WPP--NGGKLLAAEFVDPQEVKQH---
D8RWQ6_432_508         ------LRIDRFVRPFTLKALKDLLA-DTG----------N-F----T--DFWMDQIKTHCYV---------TYSSVDEAVATRNALHNLK-WPV--IGGKQLVAEFVEPEEV------
A0A0K9PH74_440_521     ----NSLRIDNFLRPFTLKAVQELLG-KTG----------T-V----C--SFWMDNIKTHCYV---------TYSSEEEAVKTRDAVYNLQ-WPA--NGGRLLMADFTDPEQVKKH---
A0A0M3JWR1_592_676     ---NQVLMIRQLTRPFTSKQLQQMLA-TYG----------T-I-V-EN--GFWIDNIKSTCIV---------KYSTIEEAIVARGRLHNVV-WPI--HSPKALKVDYSDDEGLAKH---
A0A0C2MY96_112_190     -----YVHVSNLVRPFTIGDLQEFLS-EGE----------G-L-D-TD--HFWIDSIKSHCYV---------KYKSIESAAKVVERCNGVQ-WPD--HNPKRLVVDFADIER-------
J9B3J9_106_191         --VNQVIMIRQLTRPYTVKQLQQLLS-TFG----------T-V-V-EG--GFWIDNIKSTCIA---------KYSTVEEAVVARGRLHNVV-WPP--CSPRTLKIDYSDDANLTKH---
A0A1D1XQ91_216_295     ------LRIDRFLRPFTLKAVQELLA-KTG----------T-V----C--SFWMDHIKTHCYV---------TYSSVEEAIATRNVLYNLQ-WPP--NGGRLLTADFVDPQEVKMH---
A0A176W2N2_556_631     -------KIDRFVRPFTIKAVKELLE-QTG----------T-V----K--DMWMDQIKTHCFV---------TYSAVEEAVATRNALYHLQ-WPV--AGGKLLVAEFVEPEEV------
A0A1E5WC87_606_682     ------LRIDRFVRPFTLKAVQELLG-KTG----------S-I----C--SFWMDDIKTHCYV---------TYSSVEEAVTTRDAVYNLQ-WPP--NNHNYLVAEFVDPQEV------
UPI00098DBDAD_514_593  ---TNSLRIDRFLRPFTLKAVQELLG-KTG----------N-V----S--SFWMDQIKTHCYV---------TYSSVEEAIETRNAVYNLQ-WPP--NGGRLLVAEYVDPEEV------
A0A251S9X5_413_489     ------LRIDRFLRPFTLKAVQELLG-KTG----------T-V----V--SFWMDQIKTHCYV---------TYSSVEEAIETRNAVYNLQ-WPT--NGGRLLIAEFVDPSEV------
A0A022Q8V8_68_147      ---SNSLRIDRFLRPFTLKAVQELLG-KTG----------T-V----T--SFWMDQIKTHCYV---------SYSSVEEALETRNAVYNLQ-WPP--NGGRLLLAEFVDPQEV------
A0A1U8NTI2_513_589     ------LRIDHFLRPFTLKAVQELLG-KTG----------T-V----T--SFWMDHIKTHCYV---------TYSSVEEAIETRNAVYNLQ-WPP--NGGRLLVADFVDPQEV------
A0A0N4UCY2_5_81        -----VIIIRQLTRPFTVKQLQQLLS-KFG----------Q-I-A-EN--GFWIDNIKSMCIV---------KYSSVEEAVIARGRLHNVV-WPS--CSPRMLKVDYTDE---------
A0A0N5D521_435_520     --VNQVIMIRQLTRPYTVKQLQQLLS-IFG----------T-I-V-EG--GFWIDNIKSTCIA---------KYSTVEEAIVARGRLHNVV-WPP--CSPKTLKIDYSDDANLAKH---
UPI0004E54C21_446_525  ------LRIDRFLRPFTLKAVQELLA-KTG----------T-V----C--SFWMDQIKTHCYV---------TYSSVEEAMATRNAVYNLQ-WPP--NGGKLLVAEFVDPQEVKMH---
UPI00098E3031_571_647  ------LRIDHFLRPFTLKAVQELLA-KTG----------T-V----V--DFWMDQIKTHCYV---------TYSSVDEATATRNALYNLQ-WPT--NGGRLLVAEFVEPQEV------
A0A0E0LNK8_711_787     ------LRIDRFVRPFTLKAVQELLG-KTG----------S-V----C--SFWMDHIKTHCYV---------TYSSVEEALATRNAVYNLQ-WPP--NNGSYLVAEFVDPHEV------
A0A0P6IPW8_48_129      --TSKVLFITNLVRPMTTVALRSLLS-RTG----------T-V----E--DLWIDSIKSKCFV---------IFSTEDQAYETRQALHGVK-WPE--HSLKTLNVEFSTEEEMN-----
V4UPD6_523_602         ----NSLRIDHFVRPFTLRAVQELLG-KTG----------T-F----T--SFWMDHIKTHCYV---------TYSSVEEAIETRNALYNIQ-WPP-NNGGRPLVAEFVDPQEV------
A0A0D2WU61_161_233     -----TLHIANFSRPLTLNQVNELLA-RTG----------K-V-V-PD--TFWMDSIKSQCYA---------TFETVEEANATFEALNGIN-WPA--HNMKKLVVQ------------
UPI00078901DB_534_612  ----NSLRIDRFLRPFTLKQVQELLG-KSG----------S-I----T--SFWMDQIKTHCYV---------TYSSVEEAIETRNAVYNLQ-WPP--NGGRLLVAEFVDPQEV------
UPI0007AF0CE6_524_602  ----NSLRIDQFLRPFTLKAVQELLG-KTG----------N-V----V--SFWMDQIKTHCYV---------TYSSVEEATETRNAVYNLQ-WPP--NGGRLLVAEYVDPQEV------
A0A103XK13_529_605     ------LRIDRFLRPFTLKAVQELLG-KTG----------T-V----V--SFWMDHIKTHCYV---------TYSSVEEAVETRNAVYNLQ-WPV--NGGRLLLAEFVDPSEV------
A0A0E0D1G3_327_403     ------LRIDRFVRPFTLKAVQELLG-KTG----------S-L----C--SFWMDHIKTHCYV---------TFSSVEEAVATRDAVYDLQ-WPP--NNGNRLVAEFVDPQEV------
A0A251S6M2_394_470     ------LRIDNFLRPFTLKAVQEFLG-KTG----------T-V----V--SFWMDQIKTHCYV---------TYASVEEAIETRNAVCNLQ-WPV--NGGRLLMADFVDPQEV------
UPI000901EF53_679_757  ----NSLRIDNFVRPFTLKAVQELLG-RTG----------K-I----C--NFWMDHIKTHCFV---------KFSSVEEAIETRNAVYNLQ-WPT--NGGRLLLADFVDPQEV------
A0A1D5Y2H8_499_575     ------LRIDRFVRPFTLRAVQELLG-KTG----------S-V----C--SFWMDHIKTHCYV---------TFSSVEEATATRDAVYNLQ-WPP--NNGNYLLAEFVDPQEV------
A0A1J7H743_502_578     ------LRIDRFLRPFTLKAVQELLG-KTG----------K-V----N--SFWMDQIKTHCYV---------TYSSIEEAIETRNAVYNLQ-WPP--NGGRLLVAEYVEPEEV------
G7LAF9_517_595         ----NSLRIDRFIRPFTLKAVQELLG-KSG----------N-I----I--SFWMDQIKTHCYV---------SYSTVEEATETRDAVYNLQ-WPP--KGGRLLVAEYVEPQEV------
UPI00077659FE_466_542  ------LRIDRFVRPFTLKAVQELLG-KTG----------S-V----C--SFWMDHIKTHCYV---------TYSSLEEAVATRDAVYNLQ-WPP--NNGNRLVAEFVDPQEV------
UPI000B8C7FA6_550_627  ----NSLRIDRFLRPFTLKAVQELLG-KTG----------T-A----T--SFWMDHIKTHCYV---------TYSSVEEAVETRNAVYNLQ-WPP--NGGRLLVAEFVDPQD-------
A0A1S2YBQ1_540_618     ----NSLRIDQFLRPFTLKAVQELLG-KTG----------C-V----S--SFWMDQIKTHCYV---------TYSSIEEATETRNAVYNLQ-WPP--NGGRLLVAEYVDPEEV------
UPI0002C31E11_542_620  ----NSLKIDHFLRPFTLKAVHELLG-KTG----------K-V----T--DFWMDQIKTHCFV---------TYSSVEEAVETRNGVYNLQ-WPT--NGGRLLVADFVDPQEV------
A0A0N4VIW8_206_284     ---SEYLLIRQLTRPFTVKQLKAMLG-NYG----------A-L-D-EQ--GFWIDNIKSKCIV---------KYSSKEEAVVARGSLHNLV-WPV--GNPKELKVDFIDE---------
UPI00053B04C9_466_544  ----NSLRIDRFLRPFTLKAVQELLG-KTG----------N-V----T--SFWMDNIKTHCYV---------SYSSVEEAAATRQAVYNLQ-WPP--NGGRFLTAEFVGPEEV------
A0A1U8GE11_598_676     ---TNSLKIENFVRPFTLKAVQELLA-RTG----------E-V----C--SFWMDQIKTHCYV---------TYSSVEEAIETRNAVYNLQ-WPP--KGGRLLVTDFVDPQQ-------
B8LLW2_463_541         ------LRIDRFLRPFTLKAVQELLA-KTG----------V-V----T--SFWMDHIKTHCYV---------TYSSIEEAVATRNTLYNLQ-WPS--NGGRQLIAEYVDPEEVKH----
A0A0F5CSK2_50_131      ----TVVHMRCLVRPFTLPQLRAEIG-KHG----------S-I----KDGRFWIDPLKSHCIF---------EMETVDQATSVCGALHGQH-WPP--ANPKTLSVNYSTTEDLD-----
A0A0K9PIS0_452_533     ----NSLRIDRFLRPFTLKAAQELLG-KTG----------T-V----C--SFWMDTIKTHCYV---------TYSSEEEAVETRNAIYNLQ-WPP--NGGRLLVAEFVDPQEVKKH---
W9RB93_521_599         ----NSLRIDRFLRPFTLKAVQELLG-KTG----------N-I----T--SFWMDHIKTHCYV---------TYSSVEEAIETRNAVYNLQ-WPP--NGGRLLLAEFVDPQEV------
M8BM86_648_722         ------LRVDRFVRPFTLKAVQALLG-RTG----------S-V----C--SFWMDDIKTHCYV---------TYSSVDEAVATRNAVYNLQ-WPL--NNGSYLAAEFVDPL--------
G7J4D9_527_605         ----NSLRIDQFLRPFTLKAVQELLG-KTG----------S-V----S--SFWMDQIKTHCYV---------TYSSTEEAIETRNAVYNLQ-WPT--NGGRLLIAEFVDPEEV------
UPI00057B5482_500_580  --LTNSLRIDRFLRPFTLKAVQELLG-KTG----------S-V----T--SFWMDHIKTHCYV---------TYSSVEEATETRNAVYNLQ-WPP--NGGRLLVAEFVDPQEV------
W1NWH5_494_570         ------LRIDRFLRPFTLKAVQELLA-KTG----------T-F----C--SFWMDHIKTHCYV---------TYSSVEEAVETRNALYNLQ-WPP--NGGRLLTAEFVEPEDV------
UPI000901E0A4_438_520  ---TNSLRIDRFLRPFTLKAVQELLG-KTG----------T-V----T--SFWMDNIKTHCYV---------SYSSVEEANETRNALYNLQ-WPS--NGGRLLVAEFVDPLEVKMH---
A0A067LQN5_510_588     ----NSLRIDRFVRPFTLKAVQEFLG-KTG----------T-I----T--SFWMDHIKTHCYI---------SYSSVEEAVETRNAVYNLQ-WPP--NGGRLLVAEFVDPHEV------
V4LRJ7_462_541         ---TNSLRIDHFLRPFTLKAVQELLG-KTG----------N-V----V--SFWMDHIKTHCYV---------SYSSVEEAAATREAVYNLQ-WPP--NGGRLLTAEFVGPEEV------
K4A6D5_530_606         ------LRIDRFVRPFTLKAVQELLG-KTG----------S-V----Q--NFWMDHIKTHCYV---------TFSSVDEAVATRDAVYNLQ-WPP--NNGNKLIAEFVDPQEV------
A0A0K9QK57_566_646     ---TNSLRIDRFLRPFTLKAVQELLA-KTG----------T-V----T--SFWMDHIKTHCYV---------TYSAVEEATETRNAVYNLQ-WPP--NGGRQLTAEYVDPEEVQ-----
UPI00053C910B_462_540  ----NSLRIDRFLRPFTLKAVQELLG-KTG----------N-V----A--SFWMDHIKTHCYV---------SYSSIEEATATRNAVYNLQ-WPP--NGGRLLVAEFVDPEEV------
A0A199VMY1_473_549     ------LRIDRFVRPFTLKAVQELLA-KTG----------S-V----C--SFWMDHIKTHCYV---------TYSSVEEAAATRDAVYNLQ-WPP--NNGNHLIAEFVDPNEV------
A0A0U9HKG1_390_470     -----SVRVNNLVRPFTERALRALLS-ETG----------T-I-E-EN--GFWMDSIKTHCFV---------MFKSVEDAVRTREALFNLQ-WPP--LGGKHLVVSFVAESEVE-----
```

```
                       1008        1020     1030                1040       1050 .          1060     1070   :*    1080     1090    1098
UPI00053C4F72_480_558  ----NSLRIDRFLRPFTLKAVQELLG-ETG---------N-V----T--SFWMDQIKTHCYV--------SYSSVEEAMATRNAIYSLQ-WPP--NGGRLLVAEFVGPEEV------
A0A0A8Z6E8_731_805     ------LRIDRFVRPFTLKAVQELLG-KTG---------S-V----C--SFWMDHIKTHCYV--------TYSSVEEAVDTRNAVYNLQ-WPP--NNHSCLVAEFVDPL--------
UPI0004989003_547_625  ----NSLRIDRFLRPFTLKAVQELLG-KTG---------S-V----N--DFWMDHIKTHCYV--------TYSSVEEALETRNAVYNLQ-WPP--NGGRLLAAEFVDPQEV------
A0A0N5AI75_685_760     ------LLIRQLTRPFTVKQLKGMLS-NYG---------T-L-V-ED--GFWIDNIKSKCIV--------KYSSKEEATVARGSLHNLV-WPF--GNPKELKVDFIDE---------
A0A1U8JGY7_357_435     -----SLRVDNFLRPFTLKAVQQLLG-KTG---------I-I----T--SFWMDHIKTHCYV--------TYLSVEEAIETRNAIYNLQ-WPP--NGGRLLVAEFVDPHEVE-----
UPI00077EBE04_536_614  ----NSLRIDRFLRPFTLKAVQELLG-KTG---------K-V----T--NFWMDHIKTHCYV--------TYSSVEEAIKTRNAVYNLQ-WPP--NGGRLLVAEFVDAQEV------
UPI000B9384ED_577_655  ----NSLRIDRFLRPFTLKAVQELLG-KTG---------N-V----T--SFWMDHIKTHCYV--------TYSSVEEAMKTRDAVYNLQ-WPP--NGGRLLMAEFVDPQEV------
A0A200QW37_535_613     ----NSLRIDRFLRPFTLKAVQELLA-KTG---------T-V----S--SFWMDHIKTHCYV--------TYSSVEEAKETRNALYNLQ-WPA--NGGRLLVAEFVDPQEV------
A0A1I8BWY4_454_534     ----RWVHIRGLKRPYTQRALLELLG-KFG---------T-V-D-EG--AFWIDSIKSNCIV--------TFDTVEQAELARERLHNVI-WPT--ASTDALCVEFSTDEKL------
A9TNC4_510_585         -------KIDKFLRPFTFKAVKELLA-QTG---------T-V----E--DVWMDQIKTHCYV--------TYSSVEEATATRNALYNLQ-WPP--QGGRLLTAEFVDPSEV------
UPI00084AE5FC_3008_30  ---SCVVIIKNLTRPFTLRLLQTLLQ-RTG---------R-I-VQPD--GFWVNNVKSVCIA--------RFENEDQAEETRAHLHGIQ-WPS--SNPKTLFVDFTTQEELERH---
M1CMR4_684_759         ------LKIENFLRPFTLKAVQELLA-RTG---------E-V----C--CFWMDQIKTHCYV--------TYSSVEEATETRNAVYNLQ-WPP--NGGRLLVADFVDPQQ-------
UPI000B78E54B_493_572  ---TNSLRIDRFLRPFTLKAVQELLS-KTG---------T-V----T--SFWMDHIKTHCYV--------TYSSIEEAMETRNAVYNLQ-WPP--NGGRQLLAEFVDPEEV------
A0A166G2E4_508_585     ------LRIDRFLRPFTLKAVQELLG-KTG---------T-V----T--KFWMDHIKTHCYV--------NFSSIEEAIETRNAVYNLQ-WPA--NGGRLLVAEFVEPQEVQ-----
A0A0D3BJI6_442_518     ------LRIDRFLRPFTLKAVQELLG-KTG---------N-V----T--SFWMDSIKTHCYV--------SYSSVEEAAATREAVYNLQ-WPT--NGGRLLTAEFVGSEEV------
M0SA51_147_223         ------LRIDKFLRPFTLKAVQELLA-KTG---------T-V----C--SFWMDHIKTHCYV--------TYSSVEEANATRNAVYNLQ-WPP--NGGNLLIAEFVDPQEV------
A0A0Q3NDB0_758_822     ------LRVDRFVRPFTLKAVQELLG-RTG---------S-V----C--GFWMDHIKTHCYV--------TYSSVEEAVATRNAVYNLQ-WPP--NNVP------------------
UPI0005817327_575_653  ----NSLRIDRFLRPFTLKAVQELLG-KTG---------N-V----T--SFWMDHIKTHCYV--------SYSSVEEAVETRNAVYNLQ-WPL--NGGRLLVAEFVDPQEV------
E0CRG0_576_654         ----NSLRIDRFLRPFTLKAVQELLA-KTG---------N-V----C--SFWMDHIKTHCYV--------TYSSVEEAKETRNALYNLQ-WPP--NGGRLLVAEYVDPQEV------
M0SGK0_433_508         ------LRIDRFLRPFTLKAVQELLA-KTG---------T-V----C--SFWMDHIKTHCYV--------TYSSVEEATATRNAVYNLQ-WPP--NGGNLLVAEFVDPQD-------
F6H4N5_465_543         ----NSLRIDRFLRPFTLKAVQELLG-KTG---------S-V----T--SFWMDHIKTHCYV--------SYASVEEAMETRNAVYNLQ-WPS--NGGRLLVAEFVDPQEV------
A0A166BQ88_546_623     ------LRIDRFLRPFTLKAVQELLG-KTG---------K-V----T--NFWMDHIKTHCYV--------SYSAIEEAVETRNAVYNLQ-WPA--NGGRLLVAEFVEPQEVQ-----
A0A200PYK4_615_691     ------LRIDHFLRPFTLKAVQELLA-KTG---------A-V----C--SFWMDHIKTHCYV--------MYSSVEEAIETRNALYNLQ-WPP--NGGKLLVAEFVEPQEV------
M5W712_461_539         ----NSLRIDRFLRPFTLKAVQELLG-KTG---------K-V----T--DFWMDHIKTHCYV--------TYSSAEEALETRNAVYNLQ-WPP--NGGRLLVAEFVDPQEV------
A0A0M3HTV5_169_257     ----LVVMIRQLTRPFTSERLKRMLS-TFG---------T-I-V-EG--GFWIDTIKSTCIVKVIFTVYLSYSSIEEAVAARDALHNTF-WPH--GNPKELKVDYSNESEL------
A0A0D3A0Z8_365_443     ----NSLRIDRFLRPFTLKAVQELLG-KTG---------N-V----T--SFWMDTIKTHCYV--------SYGSVEEAAATREAVYNLQ-WPP--NGGRLLTAEFVGPEEV------
UPI0009F1D314_650_726  ------LRIDNFLRPFTLKAVQELLA-QTG---------N-V----C--DFWMDHIKTHCYV--------TYSSVEEAAETRKAVYNIQ-WPP--NGGRLLAAEFVDPQEV------
W1NFM1_563_639         ------LRIDQFLRPFTLKAVQELLA-KTG---------T-V----S--KFWMDHIKTHCYV--------TYSSEDEAMATRNALYNLQ-WPS--NGGRCLTAEFVDPEEV------
A0A1U8AMV4_457_533     ------LRIDRFLRPFTLRAAQELLA-KTG---------N-V----C--SFWMDHIKTHCYV--------TYSSVEEAIETRNALYNLQ-WPS--NGGRLLVAEFVDPQEV------
O65655_457_535         ----NSLRIDRFLRPFTLKAVQELLG-KTG---------N-V----T--SFWMDHIKTHCYV--------SYPSVEEAAATREAVYNLQ-WPP--NGGRHLIAEFVRAEEV------
A0A1S4BES0_477_551     ----NSLLIGNFLRPFTLKAVQELLS-KTG---------T-V----T--NFWMDAIKTHCFV--------SFSSVEEAIETRNAIYNLQ-WPP--HVGKLLVAEFVD----------
A0A1V9ZU41_138_214     ------LRIDNFVRPFTLPAVKEWVQ-QVG---------N-F-VDDT--GFWIDSIKTHCYV--------TFPTLELATATRERLHGQV-WPT--PHGKVISCDYSAE---------
UPI000644D74D_221_302  --VSRTLLIVNFIRPFRVEHAKQLMS-ETG---------T-V----E--QFWMNDVRSYCFV--------TFATEEEAIATRNRLHGLV-WPE--LNRTKLSVEYSSEKEFE-----
A0A1U8A2D2_462_538     ------LRIDRFLRPFTLKAVQELLA-KTG---------K-V----C--SFWMDHIKTHCYV--------TYSSVEEATETRNALYNLQ-WPP--NGGRLLAAEFVDPQEV------
A9SKT2_533_607         -------KIDRFLRPFTFKAVKELLA-LTG---------T-V----Q--DIWMDQIKTHCYV--------TYSSVEEATATRNALYNLQ-WPP--QGGRLLTAEFVDPSD-------
A0A199V3C3_568_643     ------LRIDRFVRPFTLRAVQELLA-KTG---------S-V----C--SFWMDHIKTHCYV--------TYSSAEEAIATRNAVYNLQ-WPP--NGGNHLIAEFVDPQD-------
UPI0009051E64_682_756  ----NSLLIENFLRPFTLKAVQELLS-KTG---------T-V----T--NFWMDAIKTHCFV--------SFASVEEAIETRNAVYNLQ-WPP--HGGKLLVAEFVD----------
T0QL75_175_253         ---SCTLRIDNFVRPFTLPAVHALVQ-ADG---------S-F-VEDH--GFWIDGIKTHCYV--------TFATPEIATATRQRLHGVV-WPA--HHGKTLSVDYSA----------
UPI00053F6312_522_598  ------LRIDRFLRPFTLKAVQELLA-KTG---------T-V----T--SFWMDHIKTHCFV--------SYSSVEEATETRNAVFNLQ-WPP--NGGRQLVAEFVDPEEV------
UPI0008DCB91A_507_585  ----NSLRIDRFLRPFTLKAVQELLD-KTG---------S-V----A--SFWMDHIKTHCYV--------TYSSVEEAIETRNAVCNLQ-WPP--KGGRLLLAEFVDPQEV------
V9FJ94_173_250         -----TLRIDNFVRPFTLNAVKTLVQ-ELG---------N-F-V-ED--GFWMDAIKTHCFV--------TYPTSEIAEKTSAALNGKV-WPP--ENGRSLSVKLADHT--------
H3GQ66_190_266         ----NTLRIDNFIRPFTLNAVKALVQ-ECG---------D-F-V-ED--GFWMDAIKTHCFV--------TYSTPEIAEKTIAALNGKV-WPP--ENGRSLKITFTE----------
D0NGE4_164_243         ------LRIDNFIRPFTLNAVKALVQ-ELG---------N-Y-V-ED--GFWMDAIKTHCFV--------TYPTSEIAKKTSAALNGKV-WPP--ENGRSLRVKFADHSAME-----
A0A225WZM4_143_221     ------LRIDNFVRPFTLNAVKALVQ-ELG---------N-F-V-ED--GFWMDVIKTHCYV--------TYSSPEVAKKTSSALDGKV-WPP--ENGRALSVKFADHTAM------
UPI0009E2B69C_628_704  ------LRIDNFLRPFTLKAVQELLA-QTG---------K-V----C--DFWMDHIKTHCYV--------TYSSAEEATETRNAVYNLQ-WPP--NGGRLLAAEFVDPQEV------
A0A0V1MCH8_593_675     ---SPVIYVRCLTRPYAIPALHNILG-SFG---------S-Y-S-KK--NFWINNIKSSCLV--------KYDDIESAVKARDALHNVR-WPA--SNPKTLHVDFSTTEELE-----
A0A0P1A467_167_242     ------LRIDNFIRPFTLNAVKTLVQ-ELG---------N-F-V-EN--GFWMDAIKTHCFV--------TYPTSEIAVKTSKSLNGKV-WPP--ENGRSLQVKFVDH---------
A8XEJ9_279_360         --VSNIVHIRGLTRPFTERQLRNEIE-KHG---------GEI----T--DFWIDKVKSHCFA--------KLKTNEDAGNVRNAMHDTV-WPD--GNPKKLAIVFESEENM------
G4YV57_217_294         ------LRIDNFVRPFTLNAVKALVQ-ELG---------S-F-V-ED--GFWMDAIKTHCYV--------TYPSSDIAEKTKAALNGKV-WPP--ENGRSLKVEFVDHTA-------
A0A0B1SY92_223_307     ---SDIVHIRGLTRPFTDRALKAEIL-KSG---------GQI----V--DFWIDSVKSHCIV--------QMQSIDEAREVRLAMHNTQ-WPA--ANPKTLSVQFDTKENMERHR--
A0A024TNU6_162_241     ------IRIDNFIRPFTLNAVKAFVQ-EEA---------A-F-V-EN--GFWMDAIKTHCYV--------TYATTDAAIAARGRIYGTT-WPE--LSGRRLTVEFSAETAMD-----
A0A1I7TJZ6_288_368     --VSNIVHIRGLTRPFTERQLRNEIE-KHG---------GEI----V--DFWIDKVKSHCFA--------KFNSSENAKSVLEAMHNTV-WPD--GNPKKLAIVFETEEN-------
U5D2R9_350_411         -------RIDQFLCPFTLKAVQELLA-KTG---------T-V----S--KFWMDHIKTHCYV--------TYSSEEEAMATRNALYNIQ-WPS--NG--------------------
A0A0D8XTR4_334_418     ---SEIIHIRGLTRPFTDRALKGEIV-KCG---------GQI----V--DFWIDSVKSHCIV--------QMGSIEEAREVRNSMHNTQ-WPA--ANPKTLSVQFDTKENLERHR--
E3MGI2_299_380         --VSNIVHIRGLTRPFTERQLRNEIE-RHG---------GEI----V--DFWIDKVKSHCFA--------QLNTDADAGKVLDAMHDIV-WPD--GNPKKLAIVFDTEDNM------
W2TVR7_343_427         ---SDIIHIRGLTRPFTDRALKAEIL-KCG---------GQI----T--DFWIDSVKSHCIV--------QMRSVDEAREVRIAMHNTQ-WPT--ANPKTLSVQFDTKENMERHR--
P91156_292_377         --VSNVVHIRGLTRPFTEGQLRAEIQ-KNC---------GEI----V--DLWMDKVKSHCFV--------KLNSDADAGNVISAMNNVV-WPD--GNPKRLSIVYDTEDNLIKHR--
W4GA29_179_255         -----TIRIDNFIRPFTLNAVKAFVQ-VDA---------S-F-V-DD--GFWMDAIKTHCYV--------TYATTDAAIDARSRIYGTT-WPE--LSGRQLTVDFSTD---------
```

```
                            1008        1020      1030                    1040      1050 .               1060      1070  :*   1080      1090     1098
A0A1I7ZLM1_547_629      ---SCVLHICGLTRPLSLKQIFTLLS-QYG---------E-F-DEEQ--GFWIDNIKSNCYV--------QYKSTAEAALARYRLHNIQ-WPS--QNKKGLSVDFSTPEKL------
A0A1R2BW38_8_84         ------LHITNFVRPFTISQARSLLE-ETC---------R-I----Q--FFWMDSIKSQCFV--------TVSTAEEAEATFKALQDRV-WPI--ETGKALKVAFVPNSEL------
F4PWS4_251_332          --ITNILFIDKFVRPLKESACKDMLA-ETG---------N-I----V--DFWMNNIKSYCYV--------SYTTEDEAAKTREAVYGLV-WPP--QNRSPLTAEFVTQEEAE-----
A0A0N4XIK6_63_141       ---SEFIHIRGLTRPFTDRALKAEIM-KSG---------GQI----V--NFWIDGVKSHCIV--------QMQSIEEAREVRLAMHNTQ-WPA--ANPKMLSVQFDTKE--------
D3BGV9_265_346          --VSRTLIIDKFVRPFRIEMAKQLVS-ETG---------D-V----E--KFWMNDIRSYAFV--------TYATEEQAVNTRNALYGLV-WPP--LNRSKLIVDFSSEEEFE-----
A0A1I7S631_363_442      -----FIHVHRLRRPFTNKGLIALLK-KFG---------E-F-DENE--DFWIDAIKSNCIV--------KYSSIEEAKKARQELHNVI-WPT--GNPDCLLVDFCTEDR-------
A0A077Z7C0_697_782      ----KVVRITGLSRPFAVRTLRDTMA-LFG---------S-F-S-NT--HFWINKIRSVCIV--------QYDSLESAVNAREALHKVR-WPK--TNQHFLHVDFGCEGDVEYLNG-
A0A0R3PFR2_177_252      ---SEIVHIRGLTRPFTDRALKAEIT-KCG---------GQI----V--DFWIDSVKSHCIV--------QMNSIDEAREVRNTMHNTQ-WPA--ANPKTLSVQFD-----------
G0NJS1_257_338          --VSNIVHIRGLTRPFTERQLRGEIE-KHG---------GEI----T--DFWIDKVKSHCFA--------KLHSASDAKRVIEAMHDTV-WPD--GNPKRLAIVHETEENM------
A0A1Y1VF11_289_364      ------ILINNFVRPLIVRSVKELVA-QYG---------E-V----K--SFWMDSIKSHAYV--------TYTSIESAEAASKGIHGIK-FPE--ETGKILSVEYISEEE-------
A0A085NLJ6_629_714      ----KVVRVTGLSRPYAVRTLRDTMA-LFG---------S-F-P-ST--HFWINKIRSVCIV--------QYDSVESAVNAREALHKVR-WPK--TNQHCLHVDYGCEGDVEYLNG-
A0A1D6K4K9_538_600      --------------------VQEFLG-KTG---------S-V----Q--DFWMDHIKTHCYV--------TFSSVDEAVATRDAVYNLQ-WPP--NNGNKLVAEFVDPQEV------
Q54M16_225_304          ---SDTILISKLVRPFRVDMIETLMN-EYG---------S-V----K--NYWMNSVKSFCFV--------TYSTSEEAIKARNSLNGLV-WPP--LNRSKLIVEFSSESES------
A0A1Y2FFV9_327_404      ----DTILINNFVRPLVTRSVKELVG-KYG---------D-V----K--NFWMDSIKTHAFV--------TYATVESAEAAFKGINGIK-FPE--ETGRILSVEYISEEE-------
G7E7L4_303_375          ------LYLANLVRPFTPPQLKELLS-EHG---------E-L----Q--TFWLDAVKSHAYI--------VYASLDASKAAMQALQGLQ-WPA--GTGKELYLSYVP----------
A0A261XY08_694_762      ------ICVKNFVRPLVLGQVKELVG-QFG---------E-T----H--TVWLDSIKTHCFV--------SYNSPEHAVHAVKGLHGIK-FPK--DTGRILDA--------------
A0A024GA60_600_676      ------LRIDNFRRPFTLKSAKSLVQ-EFD---------N-Y-I-ED--GFWMDSIKTHCFV--------TFATCEAAERTREYLDAKI-WPQ--ENGVALRASFSSQT--------
G7YCX2_93_177           -KPTNIVYIRSLVRPFTVDQLRQMISDRFG---------Q-V----D--EIWLDRIKSSSLV--------RMNTPEAAAKCREGLDGSR-WPS--MNPRVLRCDFGNEALFDW----
A0A067CSV1_196_254      -----------------------LVQ-ADG---------V-F-VEDQ--GFWIDSIKTHCYV--------TFATPEIATATRQRLHGVV-WPA--HHGKALSVDYAV----------
A9URS4_226_290          ------VQVRNLKRPFTLIALKELLS-QHG---------T-I--LDN--TFMTDKVRSRCFA--------KFETPAQARATADALNGMQ-WPS--IH--------------------
F0W1N0_206_286          ------LRVDNFRRPFTLKSAKSLVQ-EFE---------N-Y-V-ED--GFWMDKIKTHCYV--------TYASCDVAERTRDYLDAKV-WPP--ENGVALKASFSSQTAAEI----
A0A0N5E1C6_619_704      ----KVVRITGLSRPFAVRNLCDTMA-LFG---------S-F-P-HS--HFWINNIRSVCLV--------QYDSAESATKAREALHNVR-WPK--TNQHCLQVDFGYEGEVEHLKG-
H2X565_112_193          --LSNIVHIRGLTRPFTERALRSVIE-KHG---------GEI----A--DFWIDKVKSHCFA--------KLNSESNAENVLKELNDTI-WPD--GNPKKLAIVFDTEENM------
UPI0002658D79_178_255   --------VNNLVRPFTLAQLTDFIS-HGN---------D-R-V-VT--DLWLDKIKSKAIA--------TFETSEMSERAREELHNLV-WPK--GSPKTIKCEFLTPDQVE-----
Q5C0Y3_20_100           ----DIVYIRFLVRPFTAEQLRQMIVTHFG---------P-V----T--DLWLDKIKSSSLI--------RLQTVEYATKCRDGLDGSR-WPS--MNPRVLRCEYASIDLFE-----
A0A0G4EPY7_113_188      --------IYGFVRPFTEKSVRSLLE-ETG---------T-I----R--RFWMDSIKTHCLV--------EFETEDDAIRTKDALTDVV-WPK--TSSCVLEPHFATQREMD-----
G4VKF3_456_537          ----DIVYIHFLVRPFTAEQLRQMVVTHFG---------P-V----V--DLWLDKIKSSSLI--------RLQTVEYAAKCRDGLDGSR-WPS--MNPRVLRCDYASTDLFEW----
A0A183VXS2_40_120       ----DIVYIRFLVRPFTAEQLRQMVTTHYG---------P-V----V--DLWLDKIKSSSLI--------RLQTVEYAEKCREGLDGSR-WPS--MNPRVLRCDYASIDLFE-----
V4A2G7_3_59             ---------------------------------------------DN--GFWINNIKSHCIA--------VYKEKESAEEARKTLHGTR-WPP--SNPKVLQVDFSTKQEFEQYR--
F4RSN0_621_697          ------LYIANLVRPLTVPQIKNMLS-EFG---------E-L----E--RFWIDSIRSHAYV--------TFSKLSSATAAYSKLHQTEIWPP--STGKLLTIIYLPVEE-------
A0A183AWG4_210_288      ---TNIIYIRSLVRPFTADQLRQMISERFG---------P-V----C--DLWLDRIKSSSLV--------RLESVETATRCREGLDGCR-WPS--MNPHTLHCDFGNDE--------
E1Z2I3_262_333          ------LRIDGFVRPFTERQVRELLS-ETG---------Q-V----L--ALWMPSIKTHCYA--------VFESKAQAEETRKATYHLQ-WPA--TNPKRLAPRFV-----------
A0A0L0HSW0_292_366      -----TLVIRNFIRPLTVQQVKELLA-SYG---------K-V----E--NFWMDKIKTHCYV--------TYENTNQAAEAFAGCDNLK-FPP--DTGKNLTVEYMTE---------
A0A068X447_243_318      --VSNLVYIRYLVRPFTQASLKSMLEKNFG---------T-L----T--ELWLDRIKSSAIA--------RFIDQETAVKCREGLDGCR-WPS--INPRVLHCEF------------
A0A167FJJ1_193_273      ---TNTLYVSNLSRPLVSSAFQEYVE-GVA--------EESA----S--YFWMDKLKTHCFV--------TFSSTEVATRARTAIHRSR-YPSGELSRKQLFADFIPE---------
A0A1V9XLR8_474_551      -------LVLNLVRPFTLGQLTDLVT-HSG---------V-R-P-IS--DLWLDRIKSKAIV--------EFVDESASQEAREELHNIV-WPK--GSPKTIKCEFLTEEEV------
A0A0X3PJR0_265_345      ----ELVYIRFLMRPFTPGQLSKMIESQFG---------K-V----R--ELWLDKIKSSAVV--------RMETQEAAKSCREGIDGCR-WPS--INPRILRCDFASEALLE-----
F2U9U3_270_349          ----RVVHVQNLKRPYTRLALRMLLQ-QHG---------T-LADGQE--VISTDSIRSQCYV--------EYTTHEGAVATARALSGLP-WPP--RHGQPLKATLVAE---------
U9T934_134_198          ----STLHIKNFVRPLTIPMVRDLLE-QFG---------R-I----E--YFWMDKIKTHCYV--------KFETSESAISARETLWHII-FPP--ET--------------------
A0A0L6VW96_407_482      ------LYISNLVRPLTVNQLRKKLS-EFG---------E-T----S--YFWIDPIRSHAYV--------TFQEESAALATYTSLHQTTAWPP--ETGKMLSLVFIPEK--------
A0A250XQQ6_256_335      ------LYIDGLMRPFTEATLRELLS-ETG---------K-V----V--DLWLPALKTHAVV--------TFDSEEGAEATRQALYLKK-WPL--GTKKVLQPRFISLEEAEKE---
K3WEJ1_39_101           -------------------QSKNHPS-HQG---------D-F-V-EN--GFWMDAIKTHCYV--------TYATKDAAIKMRESLDEVK-WPP--ESGIALSAAFSEV---------
D7FVT9_367_439          ------IRVDNFVRPFTAQQAKKLLE-EKA---------E-APVMEG--GFWMDGIKTHCYA--------TFDGKEAAERAMAALQGLQ-WPA--QSFKRLEA--------------
I0Z0F2_386_454          -------LITGFIRPFTEKQAQQKLS-ETG---------E-I----T--GFWMTKIKDRAYV--------IYATEEQAEATRQAVTGIE-WPL--GNGNSLRPK-------------
A0A158QTC5_1468_1546    ---SQMVYIRCLVRPFTAGQLSAMLEAHFG---------K-P----E--ELWLDKIKSSAIV--------RLADETVAARCREGLDGCR-WPS--INPKILHCEFASQE--------
UPI000BE13890_495_572   --------VLNLVRPFTLSQLTDLVT-HAG---------A-R-S-IS--DLWLDRIKSKAIV--------EFTDESASREAREELHNIV-WPK--GSPKTIKCEFLTEKEVE-----
A0A0C9ML24_240_310      ------LYIKGFVRPLIIKHVQELLS-KYG---------T-V----K--RFWMDSIKTHCYV--------TYETASQAKEAFNQVNGIK-FPA--DTGRLLTVGE------------
A0A075AW02_537_614      ------IGIQNLVRPFRTETLRQELG-RFG---------E-I----T--KFWIDGIKSRCLL--------SFKSANSAREAMENLNGLT-WPP--ETGKALLVNFVADEELD-----
A0A068RGL9_197_265      ---SSAIYIKGFVRPLIVRHVQELIG-KYG---------T-V----R--RFWMDAIKTHCYV--------IYESTEEAKAAFEGIDGIV-FPR--DTGKQ-----------------
A0A077WKV1_189_255      ---SSAIYIKGFVRPLIVRHVQELIG-QYG---------T-V----K--RFWMDAIKTHCYV--------IYESTEEAKAAFDGIDGIV-FPR--DTG-------------------
A0A0C4EH49_518_596      ----RSLYISNLVRPLTVPQLKKKLS-EYG---------E-I----T--YLWIDSVKSHAYV--------TFENDAAALATYSSMHQTEPWPP--ETGKMLVLVFVPDNE-------
A0A1Y1XHK7_352_427      ------ILINNFVRPLVTRTVKELVE-QYG---------E-V----K--SFWMDTIKTHAYV--------TYTKIEAAEAAFKGINGIK-FPL--ETGRILSVEYLTEKE-------
W7TKR7_251_327          -----TIRVDGFIRPFRLVAAKEMLE-ERG---------GGPLVGED--GMWMDVIKTHCYA--------TFQSEECAARARIALHKYQ-WPE---RGGVLTADFS-----------
A0A068YFU4_278_355      ---SSLVYIRCLVRPFTAAQLSTMLETHFG---------H-V----S--ELWLDRIKSTAVA--------RFSNEEVAARCREGLDGCR-WPS--INPRILQCEFASE---------
A0A1D1ZU61_75_147       -----ALRIDGFVRPFTERAVRELLD-PTG---------AEL----K--ALWMPSIKTHCYS--------VFASKAAAAAALAATAGLT-WPA--ASPKTLAPRH------------
D8UGA7_553_622          ----RALRIDGFVRPFTENQVRQFMS-QHG---------T-V----E--DLWMPTIKNYCYV--------VFAEVTHGEAAAAATNGVV-WPS--GNPTSIL---------------
I1C011_552_620          -----AIIVKGFVRPLIVRQAQELFA-KHG---------D-I----K--RFWMDSIKTHCYV--------IYGTIEEAQKAYTQVNGII-FPS--DTGRKLI---------------
A0A0L0W4X3_469_539      --LTNSLYISNLVRPYTIPQLKNKLS-EFG---------Q-I----T--YFWIDPIRSHAYV--------TYEDERSSLDSYKSMHQNQIWPP--ETGRK-----------------
```

```
                                1008        1020      1030               1040      1050 .           1060      1070   :*    1080      1090     1098
A0A158R9A6_1371_1448    ---SSLVYIRCLVRPFTAAQLATMLETHFG---------R-A----S--ELWLDRIKSTAVA--------RFSNEEVAAKCREGLDGCR-WPS--INPRILQCEFASE---------
A0A0B7MPJ3_107_177      -----AIYVKGFIRPLIIKHVQELFG-KYG---------T-V----K--RFWMDSIKTHCYV--------TYETRLQAKEAFANVNDIK-FPA--DTGRILTVG-------------
A0A1C7NMC0_299_376      ------LYVKGFIRPLIIRHVQELFS-KHG---------T-V----K--RFWMDMIKTHCYV--------IYENESEAKEAFNHINGIV-FPP--ETGRELTAGYLTQDQAE-----
A0A0F7TTQ9_354_408      ---------------------------------------KVV----K--EFWLDPIKTHCFV--------QFDDIASASRVRSAIHGTV-WPN-ERNRKSLWADFIPDDR-------
A0A1I8IAH0_392_447      ----------------------------------------------D--PVWLDRIKTRCYA--------HFNSIEEAKKVREALDNCR-WPK--GNSRALKVDFACQSEMDWAR--
A0A0A1N6S4_230_298      ----SAIIVKGFVRPLIIRQAQDLFA-KYG---------N-V----K--RFWMDSIKTHCYV--------IYETIEEAKKAYNQVNGII-FPS--DTGRKL----------------
N1PPY4_230_309          ------LYIRELLRPLQPNALREHLE-TIS--------QSAI----E--DFHLDKIRTHAFV--------LFESVDAATRVRAALHDNK-WPD-EAMRKPLWIDYIPEDKV------
A0A060TDM2_74_155       ---TTSVYICNLHRPIAIAVFRARLE-KIA--------QEEV----T--LFWISKLRTHCFV--------TLPSTEAAARVRDTFHNSG-YPD-RDSPTKLFADYIPPEK-------
A0A1F5LU82_347_410      ------------------------------SAPGT-ADSDTI----I--DFYLDSIKTHCFV--------SFTSVAAASRVRSAVHGTV-WPN-ERNRKSLWADFIPDDQI------
A0A1V6QH08_357_417      ---------------------------------GL-PGSDTI----I--DFYLDSIKTHCFV--------SFTSVAAASRVRSAVHGTV-WPN-ERNRKSLWADFIPHDKI------
A0A1X2HHM6_40_108       ---SNAIIITNLVRPLITRRVQEYVS-QHG---------T-V----K--RFFLDAIKTHAYV--------IYENNEQAVAAYRGMDGIQ-FPK--DTGRN-----------------
A0A1V6SEU1_332_416      -------YIDGLMRPLQPTALRKHLA-SLASAPGT-TDSDAI----I--DFYLDAIKTHCFV--------SFTSVAAASRVRSAVHGAV-WPN-ERNRKSLRADFIPDEK-------
X6LLK7_289_354          --------VEGFKRPLNTTKLLHRFG-EFG---------P-V----K--RFWINNVKTHCYI--------VFESVDDAVKCRTAMDGTY-YPT--DQPKIHA---------------
G0S6C9_235_323          ------LYIKNFMRPLREPVLKDYLV-ELAALPGTSPDPSCI----V--SFYLDPIRTHSFV--------QFSTVAAASRVRSALHGTV-WPN-ESNRKELWVDFIPEGKVD-----
A0A0M8P832_330_415      ------LYIDGLMRPLQPAALRKHLA-SLASTLET-TDSDAI----V--DFYLDSIKTHCFV--------SFTSLAAASRVRSAVHGTV-WPN-ERNRKNLRADFIPDEK-------
A0A139HG41_197_281      ---TRALYINNLIRPLKPEHLREHLL-AAS-----SSDDSVI----E--IFHLDKLRTHAFV--------LFSSNLVAVQVRAALHDKV-WPD-EPQRKALWVDYIPNEK-------
F0ZUS1_240_321          ------ILITKLVRPFRVDAFEKLIN-QYG---------QGT----E--HYWLNNIKSKAYV--------TFFNSNDAAIAMKSLDGLT-WPE--LNRTKLVVESSTLEESEYQK--
A0A1V6UN99_331_415      -------YIDGLMRPLQPSALRKHLA-SLASAPGT-TDSDDI----I--DFYLDAIKTHCFV--------SFTSLAAASRVRSAVHGTV-WPN-ERNRKNLRADFIPEEK-------
B6HNU5_351_415          ----------------------------LACAPGT-TDSDAI----V--DFHLDSIKTHCFV--------SFNSITAASRVRSAVHGTV-WPN-ERNRKNLRADFIPEEK-------
R7Z368_456_511          ----------------------------------------VL----E--SFFLDSIKTHCFC--------RFSSITAAARVRAALHGAV-WPP-ERTRKPLFVDFIPEEKLE-----
UPI000711CA4E_33_99     ------LYIDGFIRPFTMEEVKQLVS-RTV---------SPI-NLDE--HFFMNVAKTFCYV--------TYPSMEAARKAKEVIEETK-FPE--SF--------------------
A0A061RBR7_362_436      ------LRIDNFVRPFTKQLLEQMLS-AFG---------E-L----K--GIWLAPIKTHCYA--------IFESEEVAEKCRQGTYDLE-WPE--GHGKRLVPRFVALE--------
A0A197JFR3_359_426      -----ALTITNFVRPLVISQVKRMLS-EYG---------E-I----E--TLWLDSIKTHCYV--------VFKETAEAEKAYNHTNNMV-FPQ--ETGKAL----------------
A0A0W4ZM81_132_219      ------IYVGNFTRPLQIPQLKSYLL-DVIKDIDD-SNDDIL----K--KIWMDKFRTHAFI--------VFSTIKQAKRVREVLHNSI-WPC-EKGRKPLWVDYIPENKVD-----
A0A0G4PGV7_330_414      ------LYIDGLMRPLQPAALRKHLA-SLASTPET-TDSDAI----I--DFYLDSIKTHCFV--------SFTSLAAASRVRSAIHGTV-WPN-ERNRRNLRADFIPDE--------
A0A168D288_352_408      -------------------------------------STDII----Q--NFFLDQIRSHCFV--------TFTDVASAVRARSLLHDEV-WPN-ESMRKPLWIDFIPDDR-------
A0A135LLG5_330_415      ------LYIDGLMRPLQPAALRKHLA-SLASASGT-TDSDPI----I--DFHLDAIKTHCFV--------SFTSLAAASRVRSAVHGTV-WPN-ERNRKNLRADFIPDEK-------
S8BXU4_360_446          ------LYIRDLVRPLQESQLRTHLV-QLASKSA--DDDSVL----E--MIYLDGIKTHCFA--------IFSSVQACSRARNGVHGKL-FPN-EKNRKVLFADYVPEEKID-----
F9X577_224_309          -----ALYICNLVRPLQPSALREHLL-SIATPQS--NDAPEI----E--TFFLDRLRTHAFV--------LFSTLPAAIRARSSIHNQV-WPD-EPQRKPLFADFIPEEQ-------
UPI0001BF7357_347_434   ------LYIKNLMRPLRPDFLRQYLE-ELAAPPGSLRDPDSV----Q--DFHLDHIKTHAFV--------SFTSVAAAARARVALHGKI-WPN-ERNRKELWVDFIPPDRV------
G2Q2J2_296_363          ---------------------------ELAALPGAALDPDCL----V--RFYLDQVRTHAFV--------RFTSVAAASRVRTALHGTV-WPN-ERNRKELWVDFVPEDKV------
G9MZ12_257_346          -----AIYISNLMRPLRPDDLQNHLV-SLAATHDSNKGKDAI----A--RFYLDQIRTHAFV--------AFDSVATAQRVRTALHDTV-WPN-ESNRKALSVDFIPPERVD-----
A0A084RLV5_298_387      -----ALYISNLMRPLRPQDVRDHLS-GLATRPGDPINDDVI----S--NFFLDQIRTHAFV--------VFDSVYAASRVRTALHAHV-WPN-ESNRKALWVDFVPPEKVD-----
G1XC72_440_495          ----------------------------------------TL----E--SIYLDGIKTHCFA--------VFSTVQACSRARNGMHGKI-FPN-ERNRRVLFADYVPEDKVD-----
J4WJ00_296_352          ---------------------------------------GVI----V--QFHLDQIRTHAFV--------VLSSVQAAARVRSKLHDRV-WPN-ESNRKALCVDFVPAEKID-----
A0A225AA89_324_412      ------IYIRNLMRPLQPLNLKNHLL-SLAAPAEKQPDAEVL----L--DFFLDSIKTHCFA--------IFDSVSTASRVRSKLHGSV-WPN-ERDRKPLWVDFTPEDKVD-----
A0A0K0EYT9_475_542      ---KNVIYINNLTRPFCALQLKAMLQ-VHN---------T-ML--ED--HFHLMDKKSKCIA--------VYKNEEAATAISAVLAENK-FPQ--DE--------------------
A0A090LB46_470_536      ---KNVIYINNLTRPFCALQLKSMLQ-VHN---------T-ML--ED--HFHLMDKKSKCVA--------VYKNEEAAAAISAVLAENK-FPQ--D---------------------
A0A0N4Z9Q3_429_495      ---KNVVYINNLTRPFCTLQLKAMLQ-SHN---------T-ML--DE--HFYFMDKKSKCVA--------VYRSENAATAICEVLAENK-FPQ--D---------------------
```
